# Effectiveness and equity of vaccination strategies against Rift Valley fever in a heterogeneous landscape

**DOI:** 10.1371/journal.pntd.0013346

**Published:** 2025-07-28

**Authors:** Warren S. D. Tennant, Eric Cardinale, Youssouf Moutroifi, Simon E. F. Spencer, Onzade Charafouddine, Michael J. Tildesley, Raphaëlle Métras

**Affiliations:** 1 The Zeeman Institute: SBIDER, University of Warwick, Coventry, United Kingdom; 2 Mathematics Institute, University of Warwick, Coventry, United Kingdom; 3 School of Life Sciences, University of Warwick, Coventry, United Kingdom; 4 Communicable Disease Surveillance Centre (CDSC), Public Health Wales, Cardiff, United Kingdom; 5 Agence Nationale de Sécurité Sanitaire de l’Alimentation, de l’Environnement et du Travail (ANSES), Direction de la Stratégie et des Programmes, Maisons-Alfort, France; 6 Vice-Présidence en Charge de l’Agriculture, l’Elevage, la Pêche, l’Industrie, l’Energie et l’Artisanat, Moroni, Union of the Comoros; 7 Department of Statistics, University of Warwick, Coventry, United Kingdom; 8 Sorbonne Université, INSERM, Institut Pierre Louis d’Épidémiologie et de Santé Publique (Unité Mixte de Recherche en Santé 1136), Paris, France; Faculty of Science, Ain Shams University (ASU), EGYPT

## Abstract

Spatio-temporal variations in environmental and socio-agricultural factors create heterogeneity in livestock disease transmission risk, raising challenges in identifying populations most at risk and how this risk changes over time. Consequently, effective vaccination strategies require careful planning to achieve optimal or equitable outcomes across regions. We developed a metapopulation model for Rift Valley fever transmission in livestock across the Comoros archipelago which incorporates livestock vaccination in addition to heterogeneity in viral transmission rates and animal movements. We used the model to evaluate three vaccine allocation strategies–proportional allocation, optimal allocation for maximising total infections averted across the archipelago, and optimal allocation for more equitable outcomes across islands—under different vaccination coverage levels and animal identification scenarios. We report that (i) both archipelago-wide and island-specific strategy effectiveness were impacted by vaccination rate, allocation strategy, and animal identification approach, (ii) optimally allocating vaccines improved strategy effectiveness compared with proportional allocation but resulted in inequitable outcomes between islands, and (iii) tagging animals post-vaccination boosted overall strategy effectiveness for all vaccination rates.

## Author summary

Rift Valley fever (RVF) is a significant zoonotic haemorrhagic disease in Africa impacting human and livestock health. Heterogeneities in environmental, ecological and agricultural factors that influence disease transmission pose a substantial challenge to designing effective vaccination control measures in livestock. Our study uses a mathematical model that has been fitted to disease data to evaluate the effectiveness of different vaccine control strategies in mitigating RVF livestock infections in the Comoros archipelago—a network of four islands in the south-western Indian Ocean repeatedly affected by RVF. We find that a trade-off exists between maximising overall strategy effectiveness throughout the archipelago and ensuring equitable outcomes between islands. These findings contribute to a better understanding of the complexities of RVF control and inform the development of more effective vaccination programmes against livestock diseases.

## Introduction

Designing effective vaccination programmes against livestock disease is challenged by spatio-temporal heterogeneities in the environmental and socio-agricultural factors that influence disease emergence, spread and persistence. For many infectious livestock diseases, such as foot and mouth disease, Rift Valley fever (RVF) and *Peste des Petits Ruminants*, variation in disease transmission across space and time may be attributed to spatially clustered distributions of livestock [1,2], trade of livestock between affected regions [3,4], and differences in livestock species composition and susceptibility to disease [5,6]. For vector-borne zoonotic diseases, such as RVF, Bluetongue and Lumpy Skin disease, additional factors like the variation in the abundance and competence of different vector species to transmit the virus [7,8], seasonal fluctuations in vector populations [9,10], and the presence of wildlife reservoirs that can act as disease hosts [11,12], further complicate and limit our understanding of disease transmission. As a result, it is not immediately clear which populations are most at risk of infection and how this changes across time, raising difficulties in how to prioritise control strategies, such as vaccination, to achieve the most effective or equitable outcomes across or between regions.

Mathematical models are increasingly used for designing effective vaccination programmes against livestock diseases. These modelling approaches typically incorporate information on host demography, disease transmission mechanisms and properties of the vaccine. They may also be fitted to epidemiological data, allowing an analysis into how different vaccine strategies would perform in past or future forecasted outbreaks (for example, see [13–15]). However, previous modelling approaches for vector-borne livestock diseases, such as RVF, have focused on evaluating the impact of vaccination in theoretical settings [16–18] or spatially homogeneous regions [14,19,20], preventing a formal quantitative assessment of how vaccines should be allocated across a spatially heterogeneous region to achieve optimal or equitable epidemiological outcomes.

In this paper, we utilised the Comoros archipelago, a network of four islands in the southwestern Indian Ocean, as a case study to assess optimal vaccine allocation strategies against RVF while considering spatio-temporal heterogeneity in transmission and animal movement between regions. We extended a mathematical model that simulates livestock transmission dynamics of RVF in the Comoros archipelago [21] to incorporate a description of vaccine allocation across islands. The model was used to evaluate the effectiveness of three vaccine allocation strategies possible to implement in the field: (i) proportional allocation based on island livestock population sizes, (ii) optimal allocation to maximise total infections averted across the archipelago, and (iii) allocation to achieve more equitable epidemiological outcomes between islands. We further explored the impacts of tagging livestock post-vaccination to track their vaccine history and investigated the effects of targeting different age groups, frequency, and timings of vaccine administration.

## Methods

### Mathematical model

To assess the effectiveness of different vaccination strategies, we built on a previous metapopulation model describing the spread of Rift Valley fever virus (RVFV) infection in livestock across the Comoros archipelago [21] by including vaccination against RVFV infection.

#### Demography and infection.

**Fig 1:**
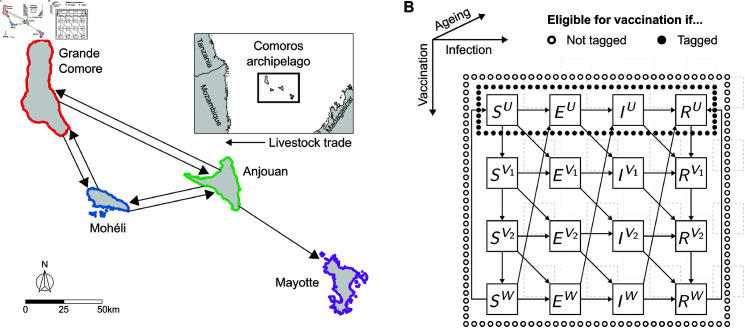
Schematic outlining the inter- and intra-island processes of livestock in the mathematical model. To evaluate the effect of vaccine control measures against Rift Valley fever virus (RVFV), we developed a mathematical model to describe RVFV infection in livestock (cattle, sheep and goats). **(A)** In the model, animals moved between the four islands in the Comoros archipelago—Grande Comore (red), Mohéli (blue), Anjouan (green) and Mayotte (purple)—as governed by the livestock trade network as shown. The administrative boundary data were made available under CC BY 3.0 [22] and CC BY-IGO [23] licences. The CC BY 3.0 licensed the data for Mayotte [24], and the CC BY-IGO licensed the data for the Comoros [25], Tanzania [26], Madagascar [27] and Mozambique [28]. All presented data was unaltered. **(B)** The livestock population of each island was divided into compartments defined by infection status, vaccination status, and age. The diagram shows the compartments (squares) and the direction of transfer of livestock between compartments (arrows). The compartments eligible for vaccination depended on whether or not livestock were tagged (black circles) or not (white circles). The notation in each compartment defines the infection status (horizontally arranged) of the animals: susceptible to the virus (*S*), infected but not yet infectious (*E*), infectious (*I*) or recovered with life-long immunity to reinfection (*R*). The superscripts denote the vaccination status (vertically arranged) of animals: unvaccinated (*U*), vaccinated with developing protection ($V_{1}$ and $V_{2}$), or vaccinated and partially protected from infection (*W*). The transitions between compartments of different age groups (diagonally arranged) are not drawn for illustrative purposes.

We modelled the livestock population as in the previous model [21]. To summarise, we used a metapopulation framework of *n* patches (for the Comoros archipelago, *n* = 4), where each patch in the metapopulation is herein referred to as an island. Here, the cattle, sheep and goat populations were modelled collectively as a single livestock population for each island. Within each island, the livestock population at each time *t* is subdivided into *A* age groups. Livestock can move between islands at different rates within the livestock trade network (Fig 1A), reflecting variations in socio-agricultural factors across the metapopulation. We also assumed that only livestock up to age group $A^{move}$ move between islands. Each age group is further divided by their infection status, namely susceptible (with compartment denoted *S*), exposed (*E*), infectious (*I*) or recovered (*R*). To account for spatio-temporal variations in disease transmission rates due to environmental and socio-agricultural factors, we incorporated the Normalised Difference Vegetation Index (NDVI) and (the natural log of) island-specific minimum disease transmission rates into the model. The proportion of animals that move from *S* to *E* per island was then modelled as an exponential function of these factors. Refer to S1 Text for a detailed description of the demographic and infection processes and S1 Table for all notation used in the mathematical model.

#### Vaccination.

Vaccination was incorporated into the model as follows, and a schematic summarising the model is shown in Fig 1B.

The vaccination status of animals is either unprotected (with compartment denoted with a superscript of *U*), first stage post-vaccination ($V_{1}$), second stage post-vaccination ($V_{2}$) or well-protected (*W*). With time, island and age group denoted using subscripts *t*, *i* and *a* respectively, the number of individuals at time *t*, island *i*, age group *a*, infection status 𝒳 and vaccination status 𝒱 is denoted by ${𝒳}_{t,i,a}^{𝒱}$. For example, the number of susceptible individuals that are not protected with a vaccine at time *t*, on island *i* and of age group *a* is denoted by $S_{t,i,a}^{U}$.

The number of vaccine doses to administer across the metapopulation per time step, ψ, is distributed to each island *i* as a proportion $\rho_{i}$. Livestock are vaccinated irrespective of their infection history, and only animals in age groups up to and including age group $A^{\text{vac}}$ were vaccinated. The rate at which livestock are vaccinated depends on whether livestock are assumed to be tagged or not. If all livestock are tagged, then the vaccination history of an animal is known, and so the vaccine is only administered to animals that were unprotected (through vaccination), *U*. If no livestock are tagged, then vaccines are administered to all livestock irrespective of their vaccination status. It is assumed that re-vaccination of livestock which are developing vaccine protection, $V_{1}$ or $V_{2}$, or vaccine-protected, *W*, does not alter their current vaccination status. The proportion of animals on island *i* of age group *a* being vaccinated at time *t* is

$$\xi_{t,i,a}=\{\begin{array}{c} \;\;\min\left\{1,\frac{\psi \rho_{i}}{\sum_{b=1}^{A^{\text{vac}}}N_{t,i,b}^{U}}\right\},\;\text{for }a\in \left\{1,\ldots ,A^{\text{vac}}\right\},\;t\geq t^{\text{vac}},\;\text{and tagged livestock,}\\ \;\;\;\;\min\left\{1,\frac{\psi \rho_{i}}{\sum_{b=1}^{A^{\text{vac}}}N_{t,i,b}}\right\},\;\text{for }a\in \left\{1,\ldots ,A^{\text{vac}}\right\},\;t\geq t^{\text{vac}},\;\text{and untagged livestock,}\\ \;\;0,\;\;\;\;\;\;\;\text{otherwise}, \end{array}$$ (1)

where $A^{\text{vac}}$ denotes the maximum age group that vaccines were administered to, and $N_{t,i,a}^{U}$ denotes the total unvaccinated population of age group *a* at time *t* on island *i*.

Livestock that are vaccinated took time to develop vaccine-induced protection. Livestock developing vaccine-induced immunity are denoted by the compartments $V_{1}$ and $V_{2}$. During this time, livestock are still infected at the same force of infection as unprotected livestock. At each time step, a proportion $p_{V_{1}\to V_{2}}$ of livestock in $V_{1}$ move to $V_{2}$, and a proportion $p_{V_{2}\to W}$ of livestock in $V_{2}$ move to the well-protected compartment, *W*. Livestock in the well-protected compartment are partially protected from infection. These livestock are infected at a proportion $\left(1-p^{eff}\right)$ of the rate that unprotected livestock are infected, where $p^{eff}$ denotes the efficacy of the vaccine.

In the model, vaccine-induced protection wanes with proportion ω. Livestock whose immunity wanes are assumed to be unprotected and eligible for re-vaccination. The probability that vaccine-induced immunity waned is given by

$$\omega =1-\exp\left(-\frac{1}{\tau_{\omega }}\right),$$ (2)

where $\tau_{\omega }$ denotes the mean number of time-steps a well-protected individual had vaccine-induced immunity to the virus.

#### Parameterisation.

Using the Comoros archipelago as a case study to evaluate the impact of different vaccine strategies against RVFV, the number of islands in the metapopulation was set to four (*n* = 4). In the model, livestock populations on each island are described on a weekly basis, where a 48-week epidemiological year (1 epidemiological week ≈ 1.08 calendar weeks) is adopted to align the model temporally to monthly empirical data. As the physical size of adult livestock relative to the size of boats used to travel between islands is large, only the first two age groups were moved between islands in the metapopulation ($A^{move}=2$). Demographic parameters were chosen to reflect the livestock population in the archipelago, while key epidemiological parameters were estimated by fitting the model without vaccination to serological data collected throughout the archipelago from July 2004 until June 2015 [21]. A detailed description of these parameters is provided in S1 Text and the values (and ranges) of all model parameters are summarised in S2 Table.

Vaccine efficacy, $p_{eff}$, and duration of protection, $\tau_{\omega }$, within the model were parameterised from the World Health Organization target product profiles for RVFV vaccines [29] and studies on existing vaccines in livestock [30–34]. For the purposes of this study to compare the effectiveness of a range of vaccination strategies against RVFV, the efficacy of the vaccine in the model was set at 90% to be in line with the target product profile for a veterinary vaccine against RVFV [29]. Trials typically follow livestock for up to two years [31–34] post-vaccination, exhibiting lasting protection for this duration. As no study to date had demonstrated protection beyond this duration, we therefore assumed that the duration of vaccine-induced protection in the model was two years. We also assumed that livestock took 2 weeks to be well-protected from infection ($p_{V_{1}\to V_{2}}=p_{V_{2}\to W}=1$) [33].

The vaccine was introduced after the end of the model fitting period ($t_{V}=528$). Six different vaccination rates, ψ, were simulated using the model, corresponding to vaccinating 5%, 10%, 15%, 20%, 25% and 30% of the total livestock population across the Comoros archipelago annually. The baseline scenario considered all age groups as eligible for vaccination ($A^{V}=10$). Sensitivity analyses were later carried out to explore the impact of focusing the same total number of vaccines on only young livestock ($A^{V}=2$). Three different approaches for allocating the vaccines to islands in the archipelago, $\rho_{i}$, were used: (i) proportional to the population size of each island (proportional allocation), (ii) optimally to maximise infections averted across the archipelago (globally optimal allocation), and (iii) optimally to maximise the infections averted on the island with the worst overall performance (equity-focused allocation). The latter two approaches of allocating vaccines are described below.

### Optimisation of vaccine allocation

One goal of the study was to determine how to distribute vaccines across the archipelago to achieve the best epidemiological outcome. Given a number of vaccines to administer in the metapopulation per unit time, ψ as described above, vaccines were allocated to each island per unit time according to the vaccine distribution ρ. We sought to optimise this distribution of vaccines by maximising some objective function that depends on ρ. Here, the objective function was defined in terms of the effectiveness of a given vaccine strategy, *g* as defined below, and included any uncertainty in model parameters (refer to S2 Table for parameter descriptors, values and ranges). Refer to the S1 Text for the full mathematical description of the objective function.

#### Vaccine strategy effectiveness.

We used two different definitions of vaccine strategy effectiveness functions, *g*_1_ and *g*_2_, to optimise the vaccine distribution across the metapopulation:

1. The first effectiveness function, *g*_1_, considered a collective approach where effectiveness was defined as the proportion of infections averted across the entire metapopulation with vaccination compared to without vaccination from the time the vaccine was first administered at $t_{v}$ up to some time *T*.
2. The second effectiveness function, denoted as *g*_2_, considered an equitable approach where effectiveness was defined as the proportion of infections averted on the worst-performing island only; that is, the island with the least number of infections averted.

The equations describing the different effectiveness functions are defined below, where the absence of any vaccination against RVFV is denoted by ${|}_{\psi =0}$ and θ denotes the model parameters.

$$g_{1}\left(\rho |\theta \right)=\frac{\sum_{i=1}^{n}\sum_{a=1}^{A}\sum_{t=t_{V}}^{T}\left[I_{t,i,a}^{U}{|}_{\psi =0}-\left(I_{t,i,a}^{U}+I_{t,i,a}^{V_{1}}+I_{t,i,a}^{V_{1}}+I_{t,i,a}^{W}\right)\right]}{\sum_{i=1}^{n}\sum_{a=1}^{A}\sum_{t=t_{V}}^{T}I_{t,i,a}^{U}{|}_{\psi =0}},$$ (3)

$$g_{2}\left(\rho |\theta \right)=\min_{i\in \left\{1,\ldots ,n\right\}}\frac{\sum_{a=1}^{A}\sum_{t=t_{V}}^{T}\left[I_{t,i,a}^{U}{|}_{\psi =0}-\left(I_{t,i,a}^{U}+I_{t,i,a}^{V_{1}}+I_{t,i,a}^{V_{1}}+I_{t,i,a}^{W}\right)\right]}{\sum_{a=1}^{A}\sum_{t=t_{V}}^{T}I_{t,i,a}^{U}{|}_{\psi =0}}.$$ (4)

#### Optimisation algorithm.

To calculate the optimal distribution of vaccines across the metapopulation for each definition of vaccine strategy effectiveness, we employed a sequential Monte Carlo (SMC) optimisation algorithm [35]. We used an SMC algorithm as it allowed us to explore potential multimodal effectiveness functions, as may be the case here with a space-time dependent model, which can be a challenge for traditional gradient-based optimisation methods. The SMC optimisation algorithm worked by gradually concentrating a set of vaccine distributions towards regions of parameter space associated with more effective vaccine strategy outcomes. Refer to the S1 Text for a detailed description of this optimisation algorithm.

### Vaccine strategies evaluated

This study aimed to assess the effectiveness of a range of long-term vaccination campaigns on disease burden in livestock, considering key factors such as vaccine allocation, age-based targeting, and the timing and frequency of vaccination. Below we detail the methods used to assess strategy effectiveness and describe the specific vaccination strategies evaluated in this study.

The effectiveness of a single vaccine strategy was calculated by first simulating the model described above forward in time (using the equations presented in S1 Text). With this approach, the model was simulated with and without vaccination up to an equivalent time horizon of June 2050 (*T* = 2207). Island-specific disease transmission rates were computed using the mean Normalised Difference Vegetation Index (NDVI) on each island from July 2004 until the end of June 2021 (see S1 Text). As NDVI data was only available for this period, for future projections, it was assumed that the observed NDVI patterns would continue, thus the existing NDVI data were replicated to cover the period up to June 2050.

For each simulation, the following metrics were calculated: (i) the percentage of infections averted across the archipelago (see Eq 3), (ii) the percentage of infections averted on each island in the archipelago, and (iii) the vaccine efficiency which was defined as the percentage of vaccines that were administered to susceptible and unprotected livestock (the ${\text{S}}^{\text{U}}$ compartment in the model).

Table 1 shows the list of vaccine strategies that were assessed in this work. The model was used to calculate the effectiveness of six vaccination strategies differing in their assumption on whether or not livestock were identifiable via tagging and vaccine allocation across the Comoros archipelago.

**Table 1:** Vaccine strategies evaluated using the mathematical model. The mathematical model describing infection with and vaccination against RVFV in livestock across the archipelago was used to assess the effectiveness of six vaccination strategies. These vaccine strategies differed in their assumption on whether or not livestock were identifiable via tagging, and how the vaccines were allocated to the four islands in the Comoros archipelago.

Livestock identifiability	Vaccine allocation
Untagged	Proportional allocation: proportional to the livestock population size of each island
Tagged	Proportional allocation: proportional to the livestock population size of each island
Untagged	Globally optimal allocation: optimised for the percentage of infections averted across the Comoros archipelago
Tagged	Globally optimal allocation: optimised for the percentage of infections averted across the Comoros archipelago
Untagged	Equity-focused allocation: optimised for the percentage of infections averted on the worst-performing island
Tagged	Equity-focused allocation: optimised for the percentage of infections averted on the worst-performing island

Three different vaccine allocations were used: (i) distributing vaccines proportional to the livestock population size of each island, (ii) optimally to maximise infections averted across the archipelago (see Eq 3), and (iii) optimally to maximise the infections averted on the island with the worst overall performance—defined as the island with the lowest percentage of infections averted in livestock (see Eq 4). Whilst the second allocation approach aimed for an optimal archipelago-wide outcome, the third allocation ensured that all islands would perform at least as well as the worst-performing island, therefore accounting for equity between island-specific outcomes. The optimisation algorithm was executed 500 times to estimate the latter two allocations. These vaccine strategies are thus herein referred to as the proportional, globally optimal and equity-focused allocations respectively.

Each vaccination strategy was evaluated with six different vaccination rates—5%, 10%, 15%, 20%, 25% and 30%—defining the percentage of livestock vaccinated across the archipelago annually.

#### Sensitivity analysis.

Finally, for each vaccination rate, we also estimated the globally optimal allocation when only the two youngest age groups were vaccinated ($A^{V}=2$), and further simulated the impact of (i) only vaccinating in the first week of each epidemiological year, and (ii) only vaccinating every two or three years.

#### Computational implementation.

The metapopulation model and optimisation algorithm were coded and executed in C++20 with the GNU Scientific Library (version 2.7) [36]. The outputs of these algorithms were analysed and visualised in R (version 4.3.2) [37] and the tidyverse library (version 2.0.0) [38]. The datasets used to simulate the mathematical model forward in time and execute the optimisation algorithm, a full description of the data, all code, including the metapopulation model, optimisation algorithm, and data analysis scripts, and a walkthrough of how to replicate our analysis for one vaccine strategy presented in our study are fully available through the GitHub repository: wtennant/rvf_vaccine [39].

## Results

The purpose of this study was to evaluate the effectiveness of different vaccine strategies against Rift Valley fever virus (RVFV). A spatially explicit, time-dependent mathematical model was developed to describe transmission of RVF in livestock across islands in the Comoros archipelago, incorporating a description of vaccination against infection (Fig 1). The model was used to investigate the effects of vaccination in the Comoros archipelago on epidemiological outcomes in livestock by estimating optimal strategies for allocation of vaccines between islands, across feasible vaccination rates, and livestock tagging strategies.

### Estimating vaccine allocation across the archipelago

**Fig 2:**
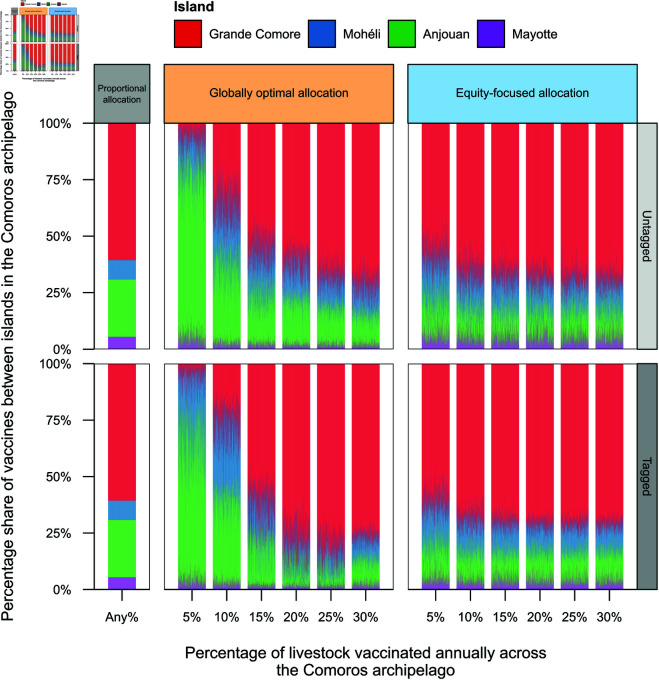
Vaccine allocation strategies across islands in the Comoros archipelago. Vaccines were allocated to each of the four islands in the archipelago—Grande Comore (red), Mohéli (blue), Anjouan (green) and Mayotte (purple)—either proportionally to the livestock population size of each island (proportional allocation; left-most panel), or optimally to maximise the percentage of infections averted across the archipelago (globally optimal allocation; middle panel), or the percentage of infections averted on the island with the worst performance (equity-focused allocation; right-most panel). Optimally allocating vaccines based on the percentage of infections averted across the archipelago was dependent on the vaccination rate, exhibiting a shift from allocating the majority of vaccines to Anjouan for lower vaccination rates, to Grande Comore for higher vaccination rates. The vertical bars show the percentage of vaccines assigned to each island for different vaccination rates and tagging strategies.

Given a number of vaccines to administer annually throughout the archipelago, vaccines were allocated to each island in three different ways: (i) proportional allocation, (ii) globally optimal allocation and (iii) equity-focused allocation. The last two allocations were estimated using an optimisation algorithm (see Methods section), which incorporated uncertainty in island-specific RVFV transmission potential and livestock movement between islands. The estimation of each vaccine allocation are shown in Fig 2, and detailed results for selected strategies are presented below. Refer to S1 Data for the numerical results for all vaccination strategies.

#### Proportional allocation: proportional to livestock population size.

By allocating vaccines proportional to the population size of each island, 60.7%, 8.6%, 25.3% and 5.4% of vaccines were allocated to Grande Comore, Mohéli, Anjouan and Mayotte, respectively, on an annual basis (Fig 2; grey, left-most panels). This vaccine allocation was independent of any vaccination rate or livestock tagging strategy.

#### Globally optimal allocation: optimal outcomes across the archipelago.

When optimising in terms of percentage of infections averted across the archipelago, the allocation of vaccines was largely dependent on the overall vaccination rate (Fig 2; orange, middle panels), and resulted in the majority of vaccines assigned to Anjouan and Grande Comore for low and high vaccination rates, respectively. These allocations were robust to both tagging strategies. For example, under the untagged scenario, vaccinating 5% of livestock across the archipelago annually favoured Anjouan, receiving a median of 71.7% (95% credible interval (CrI) = [55.4,87.2]) of vaccines, followed by Mohéli with 16.0% (95% CrI = [2.7,33.9]), Grande Comore with 6.4% (95% CrI = [0.2,20.9]), and then Mayotte with 4.0% (95% CrI = [0.3,11.1]) of vaccines. In contrast, a 30% annual vaccine coverage resulted in assigning a median of 71.9% (95% CrI = [62.6,82.4]) of vaccines to Grande Comore, 13.8% (95% CrI = [4.5,20.6]) to Mohéli, 13.02% (95% CrI = [7.3,18.3]) to Anjouan and 1.52% (95% CrI = [0.1,5.2]) to Mayotte. Similar vaccine allocations were found when animals were tagged post-vaccination. Vaccinating 5% of livestock annually in the archipelago, islands receiving vaccines from the most to the least percentage of vaccines was Anjouan (with a median of 62.8%), Mohéli (28.3%), Grande Comore (4.1%) and Mayotte (3.1%).

#### Equity-focused allocation: optimal outcomes on the worst-performing island.

In contrast to optimising in terms of infections averted across the entire archipelago, optimising vaccine allocation in terms of the percentage of infections averted on the island with the worst-performance was robust to both vaccination rate and tagging strategy (Fig 2; blue, right-most panels). With an annual archipelago-wide vaccination rate of 5%, the median (and 95% CrI) of the percentage of vaccines assigned to Grande Comore, Mohéli, Anjouan and Mayotte was 59.8% [47.1, 73.1], 18.7% [10.8, 33.3], 14.4% [6.4, 27.3], and 4.4% [1.1, 14.9], respectively, for untagged livestock; and 62.6% [49.7, 72.7], 18.1% [11.4, 30.3], 14.1% [7.1, 24.0], respectively, assuming livestock were tagged. These corresponded to a greater percentage of livestock on Grande Comore and Mohéli being vaccinated than on Anjouan and Mayotte across different vaccination rates on average. For example, vaccinating 30% of livestock in the archipelago annually and assuming animals were untagged, the median percentage of livestock vaccinated on Grande Comore and Mohéli annually was 35.1% and 43.2% respectively, whereas only 12.2% and 22.5% of livestock were vaccinated on Anjouan and Mayotte (S1 Fig).

### Assessing the effectiveness of vaccination across the archipelago

**Fig 3:**
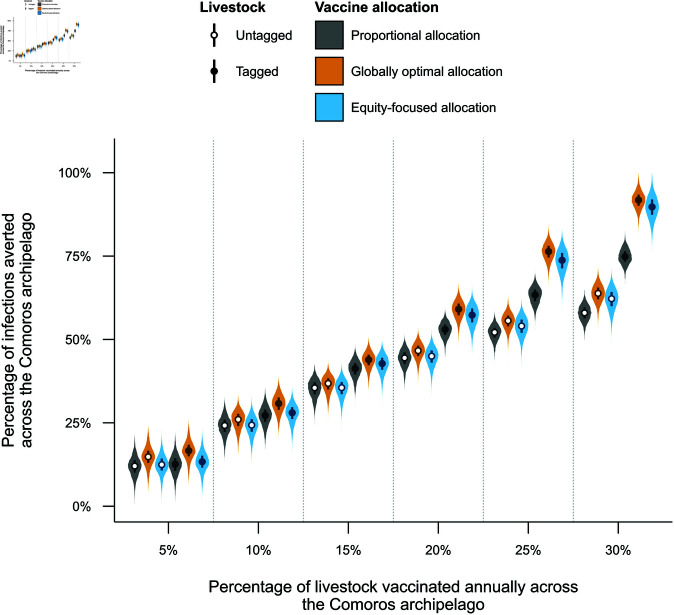
Effectiveness of different vaccine strategies against Rift Valley fever virus (RVFV) across the Comoros archipelago. For a range of vaccination rates, allocating vaccines to the four islands in the Comoros archipelago optimally by percentage of infections averted across the archipelago (orange) and by percentage of infections averted on the worst-performing island (blue; where the worst-performing island was defined as the island with the lowest percentage of infections averted) outperformed allocating vaccines proportionally to the population size of each island (grey). Tagging animals upon vaccination (black circles) also outperformed strategies where animals were not tagged (white circles), where the difference was amplified for increased vaccination rates. The violins show the percentage of infections averted across the Comoros archipelago for different annual vaccination rates, allocation methods and tagging strategies. The points and boxplots show the median and inter-quartile range for each scenario respectively. All metrics shown were based on 25,000 model simulations.

Using the three vaccine allocations as described above, the model was simulated for 35 years and compared with the model without vaccination to establish the effectiveness of a long-term continuous vaccine campaign. Fig 3 shows the percentage of infections averted over the archipelago under the different vaccination strategies.

Vaccinating a greater percentage of livestock annually resulted in a greater percentage of infections averted across the archipelago for all vaccine strategies. Allocating vaccines proportional to the population size of each island and not tagging livestock post-vaccination resulted in a median of 12.0% (95% prediction interval (PrI) = [5.9, 17.6]), 24.2% (95% PrI = [18.0, 29.5]), 35.5% (95% PrI = [29.4, 40.0]), 44.5% (95% PrI = [38.9, 48.9]), 52.1% (95% PrI = [47.2, 56.0]), 58.0% (95% PrI = [53.2, 62.2]) of infections averted when vaccinating 5%, 10%, 15%, 20%, 25% and 30% of livestock annually across the archipelago, respectively.

#### Proportional versus globally optimal and equity-focused vaccine allocations.

Both globally optimal and equity-focused vaccine allocations (Fig 3; orange and blue violins) outperformed proportional allocation (Fig 3; grey violins) for all vaccination rates and tagging strategies, with the advantage of globally optimal and equity-focused vaccine allocations increasing as the vaccination rate increased. For example, at 5% vaccination and with untagged livestock, the globally optimal allocation averted a median of 14.8% (95% PrI = [8.5, 20.8]) infections compared to the 12.0% of infections with proportional allocation. This difference widened up to a vaccination rate of 30%, with globally optimal and proportional vaccine allocations averting 63.8% (95% PrI = [58.5, 68.5]) and 58.0% of infections respectively. The percentage of infections averted across the archipelago with the globally optimal vaccine allocation (Fig 3; orange violins) consistently outperformed the equity-focused vaccine allocation (Fig 3; blue violins) in terms of infections averted across the archipelago.

#### Tagging livestock post-vaccination.

Livestock tagging post-vaccination increased the percentage of infections averted across the archipelago when compared with strategies where livestock were not tagged, for all vaccine allocations and vaccination rates, but became more pronounced as vaccination rates increased. For instance, at the largest vaccination rate (30%) and globally optimal allocation, tagging animals averted 44% more infections than not tagging animals, specifically averting a median of 62.2% (95% PrI = [55.4, 67.6]) and 89.7% (95% PrI = [82.71, 96.1]) of infections across the archipelago in the untagged and tagged scenarios, respectively.

#### Vaccine administration efficiency.

In addition to increasing infections averted, tagging livestock post-vaccination improved the efficiency of vaccine administration, defined as the proportion of vaccines administered to susceptible livestock only (i.e. not to livestock with vaccine-induced nor natural protection against RVFV), as tagging ensured a greater percentage of vaccines were administered to animals that did not have any prior protection against RVFV infection. For example, the median efficiency across the archipelago was 66.9% (95% PrI = [65.5, 68.1]) and 95.8 (95% PrI = [94.2, 97.3]) under 30% annual vaccine coverage and globally optimal allocation for the untagged and tagged scenarios, respectively. However, in all strategies the proportion of livestock with natural immunity to the virus would increase following an outbreak, resulting in a drop in vaccine administration efficiency on each island (S2 Fig, S3 Fig, and S4 Fig).

#### Impact of vaccinating the young, at the start of the year and vaccination frequency.

To investigate the effect of age-targeted vaccination, we estimated the globally optimal allocation for all scenarios where only the two youngest age groups in the model (equivalent to 50% of the livestock population in the archipelago) were vaccinated. We found that this allocation of vaccines was similar to the allocation where all age groups were vaccinated (S5 Fig). However, vaccinating only the two youngest age groups showed a small increase to the median percentage of infections averted under each strategy compared with vaccinating all age groups. For example, vaccinating 20% of all livestock annually (with post-vaccination tagging) averted 58% (95% PrI = [52, 64]) of infections across the archipelago compared with 63% (95% PrI = [57, 68]) when only young livestock were vaccinated.

Using these vaccine allocation estimates, the percentage of infections averted across the Comoros archipelago was simulated for scenarios which vaccinated only in the first month of the epidemiological year (July) and only vaccinated every two or three years. Vaccinating only in the first month of the year was similar to vaccinating throughout the year (S6 Fig), however vaccinating less frequently resulted in a lower percentage of infections averted across the Comoros archipelago (S7 Fig). This result was consistent between vaccination rates; when comparing vaccinating 10% of livestock every year versus 30% of untagged livestock every three years, for example, which averted 26% (95% PrI = [20, 30]) and 22% (95% PrI = [16, 27]) of infections, respectively.

### Evaluating the equity of island-specific strategy effectiveness

**Fig 4:**
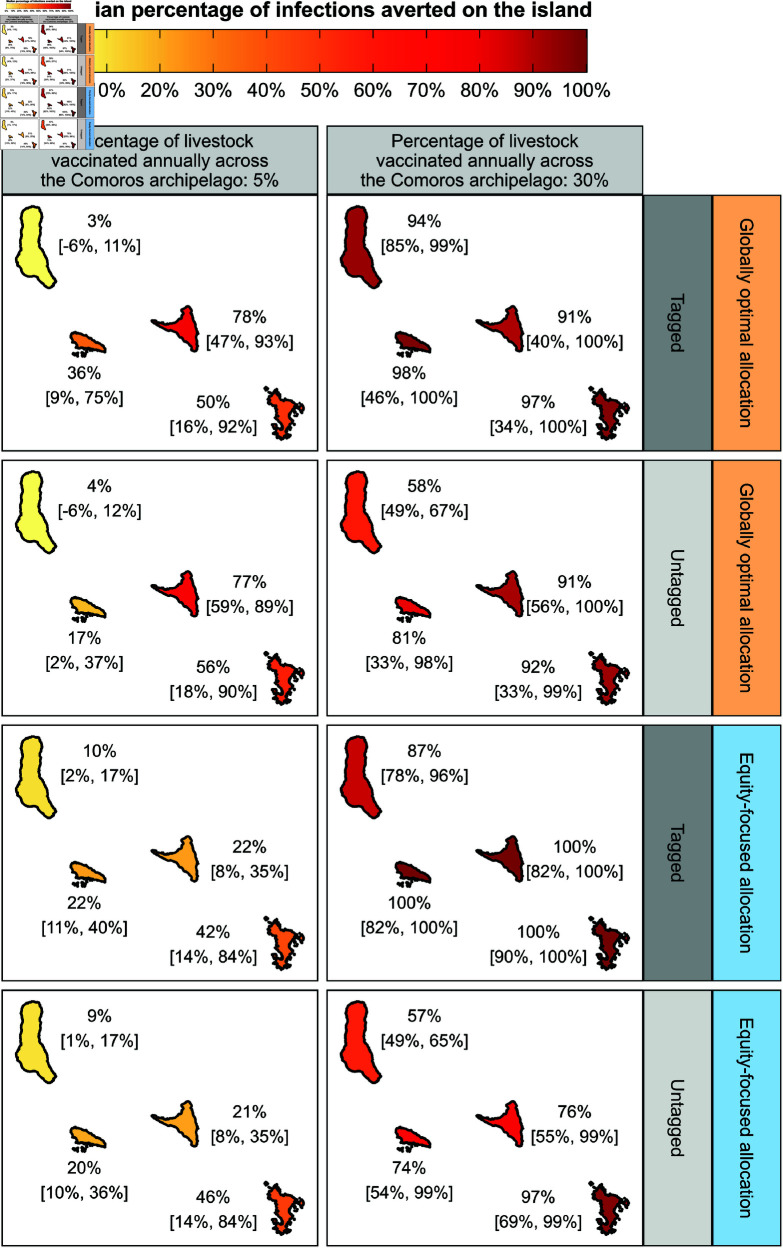
Equity in vaccine strategy effectiveness between islands in the Comoros archipelago. Optimally allocating vaccines in terms of total infections averted across the archipelago (globally optimal allocation; orange panels) resulted in an imbalance of vaccine strategy effectiveness at the island-level at low vaccination rates. To ameliorate this, vaccines were also allocated to maximise the infections averted on the worst performing island (equity-focused allocation; blue panels), resulting in a less pronounced imbalance of strategy effectiveness between islands at low vaccination rates. Shown is the median and 95% prediction interval of the percentage of infections averted on each island when vaccinating 5% and 30% of livestock across the archipelago annually under each vaccine allocation and tagging strategy. Summary statistics were generated using 25,000 model simulations. The administrative boundary data were made available under CC BY 3.0 [22] and CC BY-IGO [23] licences. The CC BY 3.0 licensed the data for Mayotte [24], and the CC BY-IGO licensed the data for the Comoros [25]. All presented data was unaltered.

To assess equity of outcomes between islands due to vaccination, the percentage of infections averted on each island was measured for all vaccination rates, vaccine allocations and tagging strategies as described above. Fig 4 shows the median and 95% prediction interval of infections averted on each island in the Comoros archipelago with the globally optimal and equity-focused vaccine allocations when vaccinating 5% and 30% of the total archipelago livestock population annually. Refer to S8 Fig for the model predicted number of infections and percentage of infections averted per island for each vaccine strategy.

#### Imbalance in strategy effectiveness between islands at low vaccination rates.

All globally optimal vaccine allocations lead to a discrepancy in the percentage of infections averted between islands, particularly at lower vaccination rates: vaccinating 5% of untagged livestock annually across the archipelago averted 4% (95% PrI = [–6, 12]) of infections on Grande Comore, 17% (95% PrI = [2, 37]) on Mohéli, 77% (95% PrI = [59, 89]) on Anjouan and 56% (95% PrI = [18, 90]) on Mayotte.

For 15 of the 36 tested combinations of vaccination rate, allocation, and tagging, at least one island had the potential to avert a negative number of infections with varying probabilities of occurrence (S8 Fig). That is, vaccination resulted in a greater number of model predicted infections than without vaccination. This was most pronounced in Grande Comore under the 5% vaccination rate and globally optimal allocation, with an estimated 18.8% risk for both the tagged and untagged livestock scenarios. In contrast, the risk of this occurrence was found to be less than 2.5% for all other vaccine strategies. These results may due to vaccination delaying seasonal outbreaks of RVFV to subsequent years where virus transmission was higher because of more suitable environmental conditions, resulting in larger outbreaks (S9 Fig, S10 Fig and S11 Fig).

#### Allocating vaccines to achieve greater equity in epidemiological outcomes.

The equity-focused vaccine allocation partially addressed the imbalance in strategy effectiveness at low vaccination rates. For example, for the same scenario (5% vaccination rate and untagged livestock), 9% (95% PrI = [1, 17]), 20% (95% PrI = [10, 36]), 21% (95% PrI = [8, 35]) and 46% (95% PrI = [14, 84]) of infections were averted on Grande Comore, Mohéli, Anjouan and Mayotte, respectively. The imbalance in infections averted on each island was also ameliorated in this manner for vaccine strategies where livestock were tagged post-vaccination.

## Discussion

Spatio-temporal heterogeneities in environmental and socio-agricultural factors present a significant challenge to developing effective vaccination strategies against infectious livestock diseases. These challenges are further amplified for vector-borne zoonotic diseases, such as RVF, where uncertainties about the role of vectors and wildlife reservoirs on disease transmission add an extra layer of complexity. Additionally, geopolitical factors can introduce pressure to balance disease control outcomes between regions while achieving maximum effectiveness globally. Given these challenges, quantifying the effectiveness of a range of vaccine allocations in a heterogeneous landscape is crucial. Therefore, our study employed a mathematical modelling approach to investigate three vaccine allocation strategies for mitigating RVFV transmission in the Comoros archipelago, a network of four islands in the southwestern Indian Ocean. Our model explicitly considers the spatial and temporal variations and uncertainty in disease transmission potential within each island, as well as the movement of livestock between islands.

Our findings suggest that (i) allocation of vaccines proportional to livestock population sizes did not lead to the most effective overall control strategy against RVFV, (ii) optimising the allocation of vaccines to achieve maximum strategy effectiveness throughout the archipelago resulted in an imbalance of strategy effectiveness at the island-level, and (iii) tracking the vaccination status of livestock substantially increased strategy effectiveness. Finally, we provided evidence that administering vaccines shortly after an outbreak was highly inefficient.

Previous modelling studies of RVF have primarily focused on mass vaccination campaigns in theoretical settings or spatially homogeneous regions, with outcomes measured over short timeframes [14,16,18–20]. Here, we evaluated the effectiveness of vaccine strategies against RVF in the context of a preventive, continuous, long-term campaign. We measured strategy effectiveness in terms of model-predicted livestock infections averted. With this approach, proportionally allocating vaccines between the four islands in the Comoros archipelago based on livestock population sizes was less effective than allocating vaccines to achieve global optimal strategy effectiveness. This was because the proportional allocation does not take into account the different transmission potential of the virus on each island. However, the globally optimal allocation was sensitive to the number of vaccines administered annually. With low vaccination rates, allocating vaccines towards Anjouan, the island with the second-lowest transmission potential but second-largest livestock population, was more effective than allocating to Grande Comore, the island with the largest transmission potential and livestock population size. This finding aligns with previous work demonstrating that focusing vaccine efforts towards a single population may be the optimal approach with limited vaccine supplies [40]. However, this could lead to significantly beneficial outcomes for one region over another, raising ethical questions over the choice between a more equitable approach or an approach maximising overall disease outcomes.

In our study, optimally allocating vaccines to maximise archipelago-wide strategy effectiveness did result in some islands experiencing a significantly lower percentage of infections averted compared to others, and this was the case for all vaccination rates. To address this, we developed an approach for maintaining high overall strategy effectiveness while achieving more equitable outcomes between islands. This approach not only improved the balance in strategy effectiveness between islands but also maintained an effective outcome globally (across the islands), outperforming proportional allocation on average. However, it is important to note that even with this approach, the equity-focused allocation did not achieve purely equal outcomes across all islands. This is likely due to the spatio-temporal heterogeneity in disease transmission and host susceptibility, which can lead to a unique set of possible qualitative outcomes for each island: frequent outbreaks (endemic), sporadic outbreaks (epidemic), or an absence of outbreaks (elimination). Therefore, it is unlikely that any vaccine allocation strategy can achieve perfectly equal epidemiological outcomes (in finite time) between regions with heterogeneous epidemiological drivers.

In contrast to the globally optimal vaccine allocation, the equity-focused allocation was less sensitive to changes in livestock vaccination rate. This suggests that an effective and equitable vaccine strategy covering regions with spatio-temporal heterogeneity in disease transmission may be devised prior to knowing how many vaccines are available, an ongoing challenge in resource limited settings [41–43]. However, even if vaccines were able to be mobilised during an epidemic effectively, our finding that the proportion of vaccines administered to susceptible livestock decreases following an outbreak, due to increases natural immunity, further highlights a potential inefficiency of a reactive vaccination approach. Additionally, we found that low vaccination rates had the potential to worsen the overall epidemiological outcomes in one island compared to no vaccination at all, underscoring an important consideration for decision-makers regarding the potential dangers of initiating a vaccine campaign with very limited vaccine supplies. Our proposal for a continuous prophylactic vaccine campaign in a resource-limited setting may motivate a reliable and sufficiently large, albeit limited, supply of vaccines ready for deployment.

In addition to availability, there are other significant barriers preventing equitable access to livestock vaccines, including acceptability, affordability and accessibility [43,44]. Some vaccines may also have existing standards on when they are administered (e.g., prior to the rainy season). Our findings suggest that strategy effectiveness may be robust to changes in the timing of vaccine administration within an epidemiological year provided it is annual. Furthermore, the cost of vaccines and farmer willingness to vaccinate are crucial factors that can significantly impact the success of any vaccination programme. The cost of vaccines can be a substantial burden for smallholder farmers, particularly in resource-limited settings. For instance, Wanyoike *et al*. [45] estimated the cost of delivery (0.67USD per head) and cost of the vaccine (0.12USD per dose) in Kenya, demonstrating the economic constraints faced by farmers or local government. Moreover, their study revealed spatial heterogeneity in farmer willingness to pay for vaccination, emphasising the importance of understanding and addressing local perceptions and attitudes towards vaccination. There may be some strategies to improve access and acceptance of vaccines through the involvement of donors and international organisations [46], however we acknowledge that there are many social, logistical and ethical barriers to effective vaccine deployment beyond what is assessed in our modelling framework.

Our work highlighted that tagging livestock in order to track their vaccine history may be beneficial for improving overall strategy effectiveness. Tagging livestock post-vaccination limits vaccine wastage—that is, our results demonstrate that focusing vaccine efforts on livestock who do not have any vaccine-induced protection against infection is beneficial not only from the perspective of the number of infections averted in livestock, but may thus reduce the cost, or demand on supply, of vaccines to achieve the same epidemiological outcomes. However, the successful implementation of a tagging programme requires careful planning to ensure the reliable and cost-effective tagging systems whilst providing adequate training, support and incentives to livestock keepers. Livestock tagging also offers broader benefits to livestock disease control beyond RVF. As demonstrated for other diseases, such as *Peste des Petits Ruminants* [13], animal identification can improve livestock disease control efforts. The tagging strategy presented here emphasises that tracking which and when livestock have been vaccinated can focus vaccine efforts towards unprotected animals. However, the assumption here is that it is (perfectly) known when vaccine-induced immunity in vaccinated livestock has waned. In reality this may only be known through serological testing, alongside a DIVA-compliant vaccine [47], to delineate whether an animal has any natural or vaccine-induced protection. A full cost-benefit analysis would be needed to determine whether it is more economically efficient for all livestock to be sampled and serologically tested for protection against RVFV prior to administration of the vaccine, or otherwise. Tagging livestock, as well as increasing overall strategy effectiveness, could help to further build the picture of the livestock trade network, which may help to address control targets for other livestock diseases, such as FMD.

Here we focused on livestock vaccination strategies, but it is important to acknowledge the significant direct and indirect impacts that effective livestock disease control can have on human health, particularly for zoonotic infections such as RVF. Reduced disease burden in livestock can directly improve food security by minimising livestock losses and ensuring a stable supply of food [48]. Furthermore, by mitigating economic losses for livestock farmers, effective livestock vaccination programmes can contribute to improved livelihoods of farmers, indirectly enhancing human health by reducing poverty and improving financial access to food [49]. While the logistical challenges of implementing a livestock vaccination programme may be greater than those associated with a human vaccination programme (in addition to the current lack of licensed human vaccine against RVFV), targeting the source of infection may provide more enduring benefits in the long-term control of RVF by disrupting transmission cycles and reducing the overall disease burden within the human-animal-environment interface.

While our study sheds light on optimal vaccine allocation for RVF control in the Comoros archipelago, some limitations in the modelling approach are important to consider. Firstly, we explicitly assumed that future trends in RVFV transmission potential over space and time (as governed by NDVI) were identical to that of the 2004–2021 period. This implies that climate effects on mosquito demography and the resulting changes in transmission rate are similar to the past when projecting vaccine strategies forward until 2050. This may not be the case given that mosquito demography is sensitive to rainfall, temperature and humidity [50–52]. However, climate forecasting was beyond the scope of our study, and instead this motivates further development of our approach to consider adaptive surveillance and control measures, whereby the optimal control strategy may be updated over time, taking into account possible changes in recent environmental predictions as well as socio-agricultural patterns (e.g., temporary movement bans). On the spatial scale, we have assumed that livestock mix homogeneously within each island. Consequently, our results imply an equitable allocation of vaccines within each island itself. This may not only be impractical, but a better approach would be to target areas of each island that are at higher risk of RVFV transmission. Directly extending our model and inference framework to finer spatial scales would require higher-resolution serological data to accurately estimate within-island livestock movements and disease transmission rates. In the absence of such data, the model could instead be adapted to incorporate assumptions about within-island heterogeneity. However, this would require reliable data on livestock densities at finer spatial scales within each island, which is currently restricted to the scale (and limitations) of the Gridded Livestock of the World [53]. Finally, we defined strategy effectiveness purely in terms of the model-predicted percentage of infections averted. In reality, a policy-maker’s evaluation of a vaccine strategy may be multi-faceted, considering not only epidemiological benefits, but also social and economical ones. As a result, the definition of an effective vaccine strategy may have multiple objectives, leading to additional trade-offs between benefits and costs, where there may not always be a clear objective optimal approach [16,54].

For the development of effective control policies, it is crucial that any vaccine-related properties considered in the model are understood in the context of any resulting recommendations. Here, we highlight how our specific choices for vaccine parameters within the model may influence the estimated vaccine allocations and the overall effectiveness of the vaccination strategies. As no study to date had demonstrated vaccine-induced protection beyond two years, we pessimistically assumed that the duration of protection was no longer than this. A target product profile for a veterinary RVFV vaccine is set to offer protection for at least 3 years [29], and this lengthened duration would likely increase the overall effectiveness of a given vaccination strategy and shift the optimal allocation of vaccines. Extended durations of vaccine-induced protection may further enhance the effectiveness of strategies targeting only the youngest livestock, as the longer-lasting protection would provide more sustained benefits. However, if livestock were to remain untagged post-vaccination, this may increase the proportion of vaccines that are administered to already vaccine-protected livestock, increasing vaccine wastage. We also chose a vaccine efficacy of 90% [29], which was implicitly assumed to be equally effective in cattle, sheep and goats. However, current vaccine profiles have shown varied immunological response between species [33], which may give rise to further heterogeneity in strategy effectiveness where livestock composition is inhomogeneous across space. Here we were limited by our simplifying assumption to model cattle, sheep, and goats as a single unit. This simplification was adopted due to limited species-specific demographic and serological data and limited reports of significant abortion events in sheep during the study period [55,56]—the most susceptible species to clinical symptoms—suggesting that local species might exhibit similar susceptibility to clinical disease as other livestock. This simplification allowed for robust parameter inference to be made given the available data, allowing us to capture key epidemiological processes and consequently evaluate the impact of vaccination strategies in the Comoros archipelago. Future studies with sufficient species-specific data may allow for a comprehensive assessment of the effect of livestock composition on optimal vaccine allocation design in an extended modelling framework.

In conclusion, our study employed a mathematical modelling approach to investigate optimal vaccine allocation strategies for mitigating Rift Valley Fever virus transmission using the Comoros archipelago as a case study. We explicitly considered the spatio-temporal heterogeneities in environmental and socio-ecological factors, demonstrating that equitable allocation across all islands may not be the most effective approach. Striving to achieve optimal outcomes over a spatially heterogeneous region may result in inequity between regions, highlighting the inherent tension between equity and effectiveness in resource allocation. However, we also identified an approach that can address some of the imbalances between competing objectives. Finally, tracking the vaccination status of livestock through tagging, thereby prioritising vaccination of susceptible animals can significantly improve epidemiological outcomes. Our study highlights the importance of considering spatio-temporal heterogeneities and trade-offs between equity and effectiveness when designing vaccine allocation strategies for livestock diseases like Rift Valley fever.

## Supporting information

S1 Text Mathematical model and optimisation algorithm. A complete description of the demographical and infection processes in the mathematical model, the full system of model equations and a full description of the sequential Monte Carlo scheme employed to determine the globally optimal and equity-focused allocations of vaccines under a range of control scenarios.
(PDF)

S1 Data Summarised numerical results for all vaccination scenarios. The file contains several spreadsheets containing the median and 95% credible and prediction intervals of several metrics calculated from simulating the mathematical model under different vaccination scenarios. The metrics summarised in this data include: the percentage of vaccines allocated to each island for the three tested vaccine allocations, the percentage of infections averted across the archipelago and on each islands, and the administration efficiency of vaccination under all tested vaccination scenarios. The first sheet is the master sheet which fully describes the seven data sheets.
(XLSX)

S1 Table Mathematical notation for livestock infection model. A mathematical model describing livestock infection with and vaccination against Rift Valley Fever virus across the four islands in the Comoros archipelago was developed. In order to determine how best to distribute a set number of vaccines across the archipelago, a Sequential Monte Carlo optimisation algorithm was used, where the objective function was the number of infections averted between July 2015 and June 2050. The table shows all mathematical notation used to describe the model.
(PDF)

S2 Table Summary of parameters used during model simulations. Model parameters were set to evaluate the impact of different vaccine strategies against Rift Valley fever virus in the Comoros archipelago. The table shows the values of model parameters used in simulating the transmission model forward in time. Some demographic parameters were fixed and chosen to reflect the livestock demography of the Comoros archipelago, and all vaccine parameters were chosen based on the World Health Organization target product profiles for vaccines and field studies on existing vaccines in livestock. The remaining parameters were estimated by fitting the model without vaccination to serological data in a Bayesian framework and are represented by the median and 95% credible interval of 10,000 posterior samples in the table. Full justification of vaccine parameters is described in the main text, with the justification for parameters for the demographic and infection processes described in S1 Text.
(PDF)

S1 Fig Animals vaccinated on each island in the Comoros archipelago for different vaccine strategies. Vaccines were allocated to each of the four islands in the archipelago either proportionally to the livestock population size of each island (grey dashed line), optimally to maximise the percentage of infections averted across the archipelago (orange violins), or the percentage of infections averted on the island with the the worst performance (blue violins). For both optimal vaccine allocations, all vaccination rates and livestock tagging strategies, the median percentage of livestock vaccinated on Mohéli was greater than the overall percentage of livestock vaccinated across the archipelago, indicating overall favour towards vaccinating Mohéli. The violins show the percentage of animals vaccinated annually on each island for different annual vaccination rates, allocation methods and tagging strategies. The points and boxplots show the median and inter-quartile range for each scenario respectively. All violins shown are based on 500 executions of the optimisation algorithm.
(PDF)

S2 Fig Efficiency of vaccine administration on each island in the Comoros archipelago at 5% and 30% vaccination rates. Shown is the weekly model predicted median (solid line) and 95% (shaded area) prediction interval of the percentage of vaccines that were administered to livestock without vaccine-induced nor natural protection against Rift Valley fever virus on each island in the Comoros archipelago—Grande Comore (red), Mohéli (blue), Anjouan (green) and Mayotte (purple)—when vaccinating 5% and 30% of livestock across the archipelago annually under both optimal vaccine allocations and tagging strategies. Summary metrics were generated using 1,000 model simulations.
(PDF)

S3 Fig Efficiency of vaccine administration on each island in the Comoros archipelago at 10% and 15% vaccination rates. Shown is the weekly model predicted median (solid line) and 95% (shaded area) prediction interval of the percentage of vaccines that were administered to livestock without vaccine-induced nor natural protection against Rift Valley fever virus on each island in the Comoros archipelago—Grande Comore (red), Mohéli (blue), Anjouan (green) and Mayotte (purple)—when vaccinating 10% and 15% of livestock across the archipelago annually under both optimal vaccine allocations and tagging strategies. Summary metrics were generated using 1,000 model simulations.
(PDF)

S4 Fig Efficiency of vaccine administration on each island in the Comoros archipelago at 20% and 25% vaccination rates. Shown is the weekly model predicted median (solid line) and 95% (shaded area) prediction interval of the percentage of vaccines that were administered to livestock without vaccine-induced nor natural protection against Rift Valley fever virus on each island in the Comoros archipelago—Grande Comore (red), Mohéli (blue), Anjouan (green) and Mayotte (purple)—when vaccinating 20% and 25% of livestock across the archipelago annually under both optimal vaccine allocations and tagging strategies. Summary metrics were generated using 1,000 model simulations.
(PDF)

S5 Fig Animals vaccinated on each island in the Comoros archipelago when targeting vaccine efforts to young livestock. Vaccines were allocated to each of the four islands in the archipelago either proportionally to the livestock population size of each island (grey dashed line), optimally to maximise the percentage of infections averted across the archipelago when vaccinating all livestock (orange violins) or targeting only young livestock (green violins). The violins show the percentage of animals vaccinated annually on each island for different annual vaccination rates, allocation methods and tagging strategies. The points and boxplots show the median and inter-quartile range for each scenario respectively. All violins shown are based on 500 executions of the optimisation algorithm.
(PDF)

S6 Fig Effectiveness of targeting vaccine efforts to young livestock and only administering vaccines in the first epidemiological month of the year. The allocation of vaccines were optimised for each vaccination rate and tagging strategy assuming that vaccines were administered across all age groups and throughout the epidemiological year. Using these vaccine allocations, the effectiveness of only vaccinating within the first epidemiological month (July) or throughout the epidemiological year were simulated using the model. Shown is the median and 95% prediction interval of the percentage of infections averted across the Comoros archipelago for each scenario. All metrics shown were based on 25,000 model simulations.
(PDF)

S7 Fig Effectiveness with altered timing and frequency of vaccine administration. The allocation of vaccines were optimised for each vaccination rate and tagging strategy assuming that vaccines were administered across all age groups and throughout the epidemiological year. Using these optimal vaccine allocation, the effectiveness of only vaccinating every two or three years, alongside only vaccinating within the first epidemiological month (July), were simulated using the model for a reduced set of vaccination rates. Shown is the median and 95% prediction interval of the percentage of infections averted across the Comoros archipelago for each scenario. All metrics shown were based on 25,000 model simulations.
(PDF)

S8 Fig Effectiveness of different vaccine strategies against Rift Valley fever virus on each island in the Comoros archipelago. For six annual vaccination rates, vaccines were allocated to islands in the Comoros archipelago either proportionally to the population size of each island (grey violins), optimally in terms of infections averted across the archipelago (orange violins) or optimally in terms of infections averted on the worst-performing island (blue). Livestock were also either tagged post-vaccination (black circles) or not (white circles). For the majority of vaccination rates, vaccine allocations and tagging strategies, Grande Comore averted the lowest percentage of infections on average (grey boxes), and tagging livestock resulted in a greater number of infections averted across the archipelago. The violins show the percentage of infections averted across on each island for different annual vaccination rates, allocation methods and tagging strategies. The points and boxplots show the median and inter-quartile range for each scenario respectively. All metrics shown were based on 25,000 model simulations.
(PDF)

S9 Fig Model predicted number of infected averted livestock per island in the Comoros archipelago with 5% and 30% vaccination rates. The fitted metapopulation model describing Rift Valley Fever virus infection in livestock on each island of the Comoros archipelago—Grande Comore (red), Mohéli (blue), Anjouan (green) and Mayotte (purple)—was simulated forward in time for 35 years from the end of the fitting period, June 2015, under a range of vaccination strategies. This was then compared with the model predicted number of infections without any vaccination over the same time period. Shown is the median (solid line) and 95% prediction interval (light area) of model predicted number of infections averted on each island for 5% and 30% of livestock vaccinated annually across the archipelago, optimal vaccine allocation across islands, and two livestock tagging strategies. The median and 95% prediction intervals were calculated from 1000 model simulations.
(PDF)

S10 Fig Model predicted number of infected averted livestock per island in the Comoros archipelago with 10% and 15% vaccination rates. The fitted metapopulation model describing Rift Valley Fever virus infection in livestock on each island of the Comoros archipelago—Grande Comore (red), Mohéli (blue), Anjouan (green) and Mayotte (purple)—was simulated forward in time for 35 years from the end of the fitting period, June 2015, under a range of vaccination strategies. This was then compared with the model predicted number of infections without any vaccination over the same time period. Shown is the median (solid line) and 95% prediction interval (light area) of model predicted number of infections averted on each island for 10% and 15% of livestock vaccinated annually across the archipelago, optimal vaccine allocation across islands, and two livestock tagging strategies. The median and 95% prediction intervals were calculated from 1000 model simulations.
(PDF)

S11 Fig Model predicted number of infected averted livestock per island in the Comoros archipelago with 20% and 25% vaccination rates. The fitted metapopulation model describing Rift Valley Fever virus infection in livestock on each island of the Comoros archipelago—Grande Comore (red), Mohéli (blue), Anjouan (green) and Mayotte (purple)—was simulated forward in time for 35 years from the end of the fitting period, June 2015, under a range of vaccination strategies. This was then compared with the model predicted number of infections without any vaccination over the same time period. Shown is the median (solid line) and 95% prediction interval (light area) of model predicted number of infections averted on each island for 20% and 25% of livestock vaccinated annually across the archipelago, optimal vaccine allocation across islands, and two livestock tagging strategies. The median and 95% prediction intervals were calculated from 1000 model simulations.
(PDF)
