# Supplementary material for: Effectiveness and equity of vaccination strategies against Rift Valley fever in a heterogeneous landscape
Source: PLoS Negl Trop Dis. 2025 Jul 28;19(7):e0013346. doi: 10.1371/journal.pntd.0013346 (PMC12316399; doi:10.1371/journal.pntd.0013346)
Supplement: S1 Text — A complete description of the demographical and infection processes in the mathematical model, the full system of model equations and a full description of the sequential Monte Carlo scheme employed to determine the globally optimal and equity-focused allocations of vaccines under a range of control scenarios. (PDF) [file pntd.0013346.s001.pdf]

S1 Text

Effectiveness and equity of vaccination strategies against Rift Valley fever in a heterogeneous landscape

Warren S. D. Tennant[1,2,3,*] Eric Cardinale[4] Youssouf Moutroifi[5]
Simon E. F. Spencer[1,6] Onzade Charafouddine[5] Mike J. Tildesley[1,2,3]
Raphaëlle Métras[7]

[1]The Zeeman Institute: SBIDER, University of Warwick, Coventry CV4 7AL, United Kingdom

[2]Mathematics Institute, University of Warwick, Coventry CV4 7AL, United Kingdom

[3]School of Life Sciences, University of Warwick, Coventry CV4 7AL, United Kingdom

[4]Agence Nationale de Sécurité Sanitaire, 94700 Maisons-Alfort, France

[5]Vice-Présidence en charge de l'Agriculture, l'Elevage, la Pêche, l'Industrie, l'Energie et l'Artisanat, B.P. 41 Mdé, Moroni, Union of the Comoros

[6]Department of Statistics, University of Warwick, Coventry CV4, 7AL, United Kingdom

[7]Sorbonne Université, INSERM, Institut Pierre Louis d'Épidémiologie et de Santé Publique (Unité Mixte de Recherche en Santé 1136), 75012 Paris, France

[*]Corresponding author: Warren.Tennant@warwick.ac.uk

Mathematical model

Below, we describe the demographic and infection processes of the mathematical model as in Tennant et al. [1]. This model was extended to include a description of vaccination (see the main text).

Demographic process

A weekly proportion of livestock, δ_a, move due to ageing from age group a to age group $a+1$, and livestock die at a weekly age-dependent proportion denoted by μ_a. The initial proportion of livestock in age group a is denoted by p_a. Livestock in age group a move from island i to island j with weekly proportion $m_{i,j,a}$, where $m_{i,i,a}$ denotes the weekly proportion of livestock of age group a remaining on island i. Only livestock up to and including age group A^{move} are moved between islands in the metapopulation. Each week, livestock are born into the first age group at a rate of ν_i and assumed to be susceptible to infection. The total livestock population on each island are assumed to be constant over time, therefore

$$\nu_i = \sum_{a=1}^{A} \left[\mu_a N_{t,i,a} + (1 - \mu_a) \sum_{j=1}^{n} (m_{i,j,a} N_{t,i,a} - m_{j,i,a} N_{t,j,a}) \right], \tag{S1}$$

where $N_{t,i,a}$ denotes the total livestock population at time t on island i in age group a.

Infection process

Susceptible livestock (S) are infected at a time- and island-dependent weekly proportion denoted by $\lambda_{t,i}$ such that

$$\lambda_{t,i} = 1 - \exp\left(\beta_{t,i} \frac{\sum_{a=1}^{A} I_{t,i,a}}{\sum_{a=1}^{A} N_{t,i,a}} \right), \tag{S2}$$

where $\beta_{t,i}$ denotes the weekly transmission rate of RVFV. As mosquitoes are not explicitly modelled, Normalised Difference Vegetation Index (NDVI) is used as a proxy to capture the effects of seasonal oscillations in mosquito demography on Rift Valley fever virus transmission rates. We assumed that the transmission rate scaled exponentially with island-specific increases NDVI, denoted by α, where the (natural logarithm of the) minimum transmission rate per island is denoted by γ_i [1]. Therefore,

$$\beta_{t,i} = \exp\left[\alpha \left(\text{NDVI}_{t,i} - \min_{s,j} \text{NDVI}_{s,j} \right) + \gamma_i \right], \tag{S3}$$

where $\text{NDVI}_{t,i}$ denotes the Normalised Difference Vegetation Index at time t on island i.

Upon infection, livestock move into the exposed compartment (E) where they remain for one week before

transitioning to the infectious compartment (I). After one week in the infectious compartment, livestock transition into the recovered compartment (R) where they are assumed to have life-long immunity to reinfection. Infectious livestock of age group a are introduced into island i from outside the metapopulation system at a time-dependent rate $I^{\text{ext}}_{t,i,a}$. External introduction of livestock begins at time $t^{\text{ext}}_{(\text{start})}$ and lasts for $t^{\text{ext}}_{(\text{duration})}$ weeks. Infectious livestock are externally introduced in this way every $t^{\text{ext}}_{(\text{freq})}$ weeks. Only infectious livestock up to age A^{ext} are imported into the metapopulation, and are assumed to be distributed in age according to the initial age distribution of livestock, p_a. Therefore,

$$I^{\text{ext}}_{t,i,a} = \begin{cases} I^{\text{ext}}_i \dfrac{p_a}{\sum_{b=1}^{A^{\text{ext}}} p_b}, & \text{for } a \in \left\{1, \ldots, A^{\text{ext}}\right\} \text{ and } t - t^{\text{ext}}_{(\text{start})} \leq t^{\text{ext}}_{(\text{duration})} \left(\bmod\ t^{\text{ext}}_{(\text{freq})}\right), \\ 0, & \text{otherwise,} \end{cases} \tag{S4}$$

where I^{ext}_i denotes the expected number of infectious livestock introduced into island i each week.

Model equations

With the demographic and infection processes as described above, and the model structure and vaccination process described in the main text, the complete set of model equations is defined as follows.

Given the number of livestock in each compartment at time t on island i and rates of transition between compartments as defined above, the number of livestock in each compartment at time $t+1$ was defined as follows: for animals in the first age group ($a = 1$),

$$\begin{aligned} S^U_{t+1,i,1} = \nu_{t,i} + \sum_{j=1}^{n} m_{j,i,1} (1-\delta_1)(1-\mu_1) \Big\{ & (1-\lambda_{t,j})(1-\xi_{t,j,1}) S^U_{t,j,1} \\ & + \left[1 - \left(1 - p^{\text{eff}}\right)\lambda_{t,j}\right] \omega S^W_{t,j,1} \Big\}, \end{aligned} \tag{S5}$$

$$E^U_{t+1,i,1} = \sum_{j=1}^{n} m_{j,i,1} (1-\delta_1)(1-\mu_1) \lambda_{t,j} \left[(1-\xi_{t,j,1}) S^U_{t,j,1} + \left(1 - p^{\text{eff}}\right) \omega S^W_{t,j,1}\right], \tag{S6}$$

$$I^U_{t+1,i,1} = I^{\text{ext}}_{i,1} + \sum_{j=1}^{n} m_{j,i,1} (1-\delta_1)(1-\mu_1) \left[(1-\xi_{t,j,1}) E^U_{t,j,1} + \omega E^W_{t,j,1}\right], \tag{S7}$$

$$R^U_{t+1,i,1} = \sum_{j=1}^{n} m_{j,i,1} (1-\delta_1)(1-\mu_1) \left[(1-\xi_{t,j,1}) \left(I^U_{t,j,1} + R^U_{t,j,1}\right) + \omega \left(I^W_{t,j,1} + R^W_{t,j,1}\right)\right], \tag{S8}$$

$$S^{V_1}_{t+1,i,1} = \sum_{j=1}^{n} m_{j,i,1} (1-\delta_1)(1-\mu_1)(1-\lambda_{t,j}) \left[\xi_{t,j,1} S^U_{t,j,1} + (1 - p_{V_1 \to V_2}) S^{V_1}_{t,j,1}\right], \tag{S9}$$

$$E^{V_1}_{t+1,i,1} = \sum_{j=1}^{n} m_{j,i,1} (1-\delta_1)(1-\mu_1) \lambda_{t,j} \left[\xi_{t,j,1} S^U_{t,j,1} + (1 - p_{V_1 \to V_2}) S^{V_1}_{t,j,1}\right], \tag{S10}$$

$$I^{V_1}_{t+1,i,1} = \sum_{j=1}^{n} m_{j,i,1} (1-\delta_1)(1-\mu_1) \left[\xi_{t,j,1} E^U_{t,j,1} + (1 - p_{V_1 \to V_2}) E^{V_1}_{t,j,1}\right], \tag{S11}$$

$$R^{V_1}_{t+1,i,1} = \sum_{j=1}^{n} m_{j,i,1} (1-\delta_1)(1-\mu_1) \left[\xi_{t,j,1} \left(I^U_{t,j,1} + R^U_{t,j,1}\right) + (1 - p_{V_1 \to V_2}) \left(I^{V_1}_{t,j,1} + R^{V_1}_{t,j,1}\right)\right], \tag{S12}$$

$$S^{V_2}_{t+1,i,1} = \sum_{j=1}^{n} m_{j,i,1}\left(1-\delta_1\right)\left(1-\mu_1\right)\left(1-\lambda_{t,j}\right)\left[p_{V_1\to V_2} S^{V_1}_{t,j,1} + \left(1-p_{V_2\to W}\right) S^{V_2}_{t,j,1}\right], \tag{S13}$$

$$E^{V_2}_{t+1,i,1} = \sum_{j=1}^{n} m_{j,i,1}\left(1-\delta_1\right)\left(1-\mu_1\right)\lambda_{t,j}\left[p_{V_1\to V_2} S^{V_1}_{t,j,1} + \left(1-p_{V_2\to W}\right) S^{V_2}_{t,j,1}\right], \tag{S14}$$

$$I^{V_2}_{t+1,i,1} = \sum_{j=1}^{n} m_{j,i,1}\left(1-\delta_1\right)\left(1-\mu_1\right)\left[p_{V_1\to V_2} E^{V_1}_{t,j,1} + \left(1-p_{V_2\to W}\right) E^{V_2}_{t,j,1}\right], \tag{S15}$$

$$R^{V_2}_{t+1,i,1} = \sum_{j=1}^{n} m_{j,i,1}\left(1-\delta_1\right)\left(1-\mu_1\right)\left[p_{V_1\to V_2}\left(I^{V_1}_{t,j,1} + R^{V_1}_{t,j,1}\right) + \left(1-p_{V_2\to W}\right)\left(I^{V_2}_{t,j,1} + R^{V_2}_{t,j,1}\right)\right], \tag{S16}$$

$$S^{W}_{t+1,i,1} = \sum_{j=1}^{n} m_{j,i,1}\left(1-\delta_1\right)\left(1-\mu_1\right)\left\{\left(1-\lambda_{t,j}\right) p_{V_2\to W} S^{V_2}_{t,j,1} + \left[1-\left(1-p^{\text{eff}}\right)\lambda_{t,j}\right]\left(1-\omega\right) S^{W}_{t,j,1}\right\}, \tag{S17}$$

$$E^{W}_{t+1,i,1} = \sum_{j=1}^{n} m_{j,i,1}\left(1-\delta_1\right)\left(1-\mu_1\right)\lambda_{t,j}\left[p_{V_2\to W} S^{V_2}_{t,j,1} + \left(1-p^{\text{eff}}\right)\left(1-\omega\right) S^{W}_{t,j,1}\right] \tag{S18}$$

$$I^{W}_{t+1,i,1} = \sum_{j=1}^{n} m_{j,i,1}\left(1-\delta_1\right)\left(1-\mu_1\right)\left[p_{V_2\to W} E^{V_2}_{t,j,1} + \left(1-\omega\right) E^{W}_{t,j,1}\right], \tag{S19}$$

$$R^{W}_{t+1,i,1} = \sum_{j=1}^{n} m_{j,i,1}\left(1-\delta_1\right)\left(1-\mu_1\right)\left[p_{V_2\to W}\left(I^{V_2}_{t,j,1} + R^{V_2}_{t,j,1}\right) + \left(1-\omega\right)\left(I^{W}_{t,j,1} + R^{W}_{t,j,1}\right)\right], \tag{S20}$$

and for livestock in the remaining age groups $a \in \{2, \dots, A\}$,

$$\begin{aligned} S^{U}_{t+1,i,a} = &\sum_{j=1}^{n} m_{j,i,a}\left(1-\delta_a\right)\left(1-\mu_a\right)\left\{\left(1-\lambda_{t,j}\right)\left(1-\xi_{t,j,a}\right) S^{U}_{t,j,a} + \left[1-\left(1-p^{\text{eff}}\right)\lambda_{t,j}\right]\omega S^{W}_{t,j,a}\right\} \\ &+ \sum_{j=1}^{n} m_{j,i,a-1}\,\delta_{a-1}\left(1-\mu_{a-1}\right)\left\{\left(1-\lambda_{t,j}\right)\left(1-\xi_{t,j,a-1}\right) S^{U}_{t,j,a-1} \right. \\ &\qquad \left. + \left[1-\left(1-p^{\text{eff}}\right)\lambda_{t,j}\right]\omega S^{W}_{t,j,a-1}\right\}, \end{aligned} \tag{S21}$$

$$\begin{aligned} E^{U}_{t+1,i,a} = &\sum_{j=1}^{n} m_{j,i,a}\left(1-\delta_a\right)\left(1-\mu_a\right)\lambda_{t,j}\left[\left(1-\xi_{t,j,a}\right) S^{U}_{t,j,a} + \left(1-p^{\text{eff}}\right)\omega S^{W}_{t,j,a}\right] \\ &+ \sum_{j=1}^{n} m_{j,i,a-1}\,\delta_{a-1}\left(1-\mu_{a-1}\right)\lambda_{t,j}\left[\left(1-\xi_{t,j,a-1}\right) S^{U}_{t,j,a-1} + \left(1-p^{\text{eff}}\right)\omega S^{W}_{t,j,a-1}\right], \end{aligned} \tag{S22}$$

$$\begin{aligned} I^{U}_{t+1,i,a} = &I^{\text{ext}}_{t,i,a} + \sum_{j=1}^{n} m_{j,i,a}\left(1-\delta_a\right)\left(1-\mu_a\right)\left[\left(1-\xi_{t,j,a}\right) E^{U}_{t,j,a} + \omega E^{W}_{t,j,a}\right] \\ &+ \sum_{j=1}^{n} m_{j,i,a-1}\,\delta_{a-1}\left(1-\mu_{a-1}\right)\left[\left(1-\xi_{t,j,a-1}\right) E^{U}_{t,j,a-1} + \omega E^{W}_{t,j,a-1}\right], \end{aligned} \tag{S23}$$

$$\begin{aligned} R^{U}_{t+1,i,a} = &\sum_{j=1}^{n} m_{j,i,a}\left(1-\delta_a\right)\left(1-\mu_a\right)\left[\left(1-\xi_{t,j,a}\right)\left(I^{U}_{t,j,a} + R^{U}_{t,j,a}\right) + \omega\left(I^{W}_{t,j,a} + R^{W}_{t,j,a}\right)\right] \\ &+ \sum_{j=1}^{n} m_{j,i,a-1}\,\delta_{a-1}\left(1-\mu_{a-1}\right)\left[\left(1-\xi_{t,j,a-1}\right)\left(I^{U}_{t,j,a-1} + R^{U}_{t,j,a-1}\right) \right. \\ &\qquad \left. + \omega\left(I^{W}_{t,j,a-1} + R^{W}_{t,j,a-1}\right)\right], \end{aligned} \tag{S24}$$

$$\begin{aligned} S^{V_1}_{t+1,i,a} = &\sum_{j=1}^{n} m_{j,i,a}\left(1-\delta_a\right)\left(1-\mu_a\right)\left(1-\lambda_{t,j}\right)\left[\xi_{t,j,a} S^{U}_{t,j,a} + \left(1-p_{V_1\to V_2}\right) S^{V_1}_{t,j,a}\right] \\ &+ \sum_{j=1}^{n} m_{j,i,a-1}\,\delta_{a-1}\left(1-\mu_{a-1}\right)\left(1-\lambda_{t,j}\right)\left[\xi_{t,j,a-1} S^{U}_{t,j,a-1} + \left(1-p_{V_1\to V_2}\right) S^{V_1}_{t,j,a-1}\right], \end{aligned} \tag{S25}$$

$$\begin{aligned} E^{V_1}_{t+1,i,a} &= \sum_{j=1}^{n} m_{j,i,a}\left(1-\delta_a\right)\left(1-\mu_a\right)\lambda_{t,j}\left[\xi_{t,j,a}S^{U}_{t,j,a} + \left(1-p_{V_1\to V_2}\right)S^{V_1}_{t,j,a}\right] \\ &+ \sum_{j=1}^{n} m_{j,i,a-1}\;\delta_{a-1}\left(1-\mu_{a-1}\right)\lambda_{t,j}\left[\xi_{t,j,a-1}S^{U}_{t,j,a-1} + \left(1-p_{V_1\to V_2}\right)S^{V_1}_{t,j,a-1}\right], \end{aligned} \tag{S26}$$

$$\begin{aligned} I^{V_1}_{t+1,i,a} &= \sum_{j=1}^{n} m_{j,i,a}\left(1-\delta_a\right)\left(1-\mu_a\right)\left[\xi_{t,j,a}E^{U}_{t,j,a} + \left(1-p_{V_1\to V_2}\right)E^{V_1}_{t,j,a}\right] \\ &+ \sum_{j=1}^{n} m_{j,i,a-1}\;\delta_{a-1}\left(1-\mu_{a-1}\right)\left[\xi_{t,j,a-1}E^{U}_{t,j,a-1} + \left(1-p_{V_1\to V_2}\right)E^{V_1}_{t,j,a-1}\right], \end{aligned} \tag{S27}$$

$$\begin{aligned} R^{V_1}_{t+1,i,a} &= \sum_{j=1}^{n} m_{j,i,a}\left(1-\delta_a\right)\left(1-\mu_a\right)\left[\xi_{t,j,a}\left(I^{U}_{t,j,a} + R^{U}_{t,j,a}\right) + \left(1-p_{V_1\to V_2}\right)\left(I^{V_1}_{t,j,a} + R^{V_1}_{t,j,a}\right)\right] \\ &+ \sum_{j=1}^{n} m_{j,i,a-1}\;\delta_{a-1}\left(1-\mu_{a-1}\right)\Big[\xi_{t,j,a-1}\left(I^{U}_{t,j,a-1} + R^{U}_{t,j,a-1}\right) \\ &\qquad + \left(1-p_{V_1\to V_2}\right)\left(I^{V_1}_{t,j,a-1} + R^{V_1}_{t,j,a-1}\right)\Big], \end{aligned} \tag{S28}$$

$$\begin{aligned} S^{V_2}_{t+1,i,a} &= \sum_{j=1}^{n} m_{j,i,a}\left(1-\delta_a\right)\left(1-\mu_a\right)\left(1-\lambda_{t,j}\right)\left[p_{V_1\to V_2}S^{V_1}_{t,j,a} + \left(1-p_{V_2\to W}\right)S^{V_2}_{t,j,a}\right] \\ &+ \sum_{j=1}^{n} m_{j,i,a-1}\;\delta_{a-1}\left(1-\mu_{a-1}\right)\left(1-\lambda_{t,j}\right)\left[p_{V_1\to V_2}S^{V_1}_{t,j,a-1} + \left(1-p_{V_2\to W}\right)S^{V_2}_{t,j,a-1}\right], \end{aligned} \tag{S29}$$

$$\begin{aligned} E^{V_2}_{t+1,i,a} &= \sum_{j=1}^{n} m_{j,i,a}\left(1-\delta_a\right)\left(1-\mu_a\right)\lambda_{t,j}\left[p_{V_1\to V_2}S^{V_1}_{t,j,a} + \left(1-p_{V_2\to W}\right)S^{V_2}_{t,j,a}\right] \\ &+ \sum_{j=1}^{n} m_{j,i,a-1}\;\delta_{a-1}\left(1-\mu_{a-1}\right)\lambda_{t,j}\left[p_{V_1\to V_2}S^{V_1}_{t,j,a-1} + \left(1-p_{V_2\to W}\right)S^{V_2}_{t,j,a-1}\right], \end{aligned} \tag{S30}$$

$$\begin{aligned} I^{V_2}_{t+1,i,a} &= \sum_{j=1}^{n} m_{j,i,a}\left(1-\delta_a\right)\left(1-\mu_a\right)\left[p_{V_1\to V_2}E^{V_1}_{t,j,a} + \left(1-p_{V_2\to W}\right)E^{V_2}_{t,j,a}\right] \\ &+ \sum_{j=1}^{n} m_{j,i,a-1}\;\delta_{a-1}\left(1-\mu_{a-1}\right)\left[p_{V_1\to V_2}E^{V_1}_{t,j,a-1} + \left(1-p_{V_2\to W}\right)E^{V_2}_{t,j,a-1}\right], \end{aligned} \tag{S31}$$

$$\begin{aligned} R^{V_2}_{t+1,i,a} &= \sum_{j=1}^{n} m_{j,i,a}\left(1-\delta_a\right)\left(1-\mu_a\right)\left[p_{V_1\to V_2}\left(I^{V_1}_{t,j,a} + R^{V_1}_{t,j,a}\right) + \left(1-p_{V_2\to W}\right)\left(I^{V_2}_{t,j,a} + R^{V_2}_{t,j,a}\right)\right] \\ &+ \sum_{j=1}^{n} m_{j,i,a-1}\;\delta_{a-1}\left(1-\mu_{a-1}\right)\Big[p_{V_1\to V_2}\left(I^{V_1}_{t,j,a-1} + R^{V_1}_{t,j,a-1}\right) \\ &\qquad + \left(1-p_{V_2\to W}\right)\left(I^{V_2}_{t,j,a-1} + R^{V_2}_{t,j,a-1}\right)\Big], \end{aligned} \tag{S32}$$

$$\begin{aligned} S^{W}_{t+1,i,a} &= \sum_{j=1}^{n} m_{j,i,a}\left(1-\delta_a\right)\left(1-\mu_a\right)\left\{\left(1-\lambda_{t,j}\right)p_{V_2\to W}S^{V_2}_{t,j,a} + \left[1-\left(1-p^{\text{eff}}\right)\lambda_{t,j}\right]\left(1-\omega\right)S^{W}_{t,j,a}\right\} \\ &+ \sum_{j=1}^{n} m_{j,i,a-1}\;\delta_{a-1}\left(1-\mu_{a-1}\right)\Big\{\left(1-\lambda_{t,j}\right)p_{V_2\to W}S^{V_2}_{t,j,a-1} \\ &\qquad + \left[1-\left(1-p^{\text{eff}}\right)\lambda_{t,j}\right]\left(1-\omega\right)S^{W}_{t,j,a-1}\Big\}, \end{aligned} \tag{S33}$$

$$\begin{aligned} E^{W}_{t+1,i,a} &= \sum_{j=1}^{n} m_{j,i,a}\left(1-\delta_a\right)\left(1-\mu_a\right)\lambda_{t,j}\left[p_{V_2\to W}S^{V_2}_{t,j,a} + \left(1-p^{\text{eff}}\right)\left(1-\omega\right)S^{W}_{t,j,a}\right] \\ &+ \sum_{j=1}^{n} m_{j,i,a-1}\;\delta_{a-1}\left(1-\mu_{a-1}\right)\lambda_{t,j}\left[p_{V_2\to W}S^{V_2}_{t,j,a-1} + \left(1-p^{\text{eff}}\right)\left(1-\omega\right)S^{W}_{t,j,a-1}\right], \end{aligned} \tag{S34}$$

$$I^W_{t+1,i,a} = \sum_{j=1}^{n} m_{j,i,a} \left(1-\delta_a\right)\left(1-\mu_a\right)\left[p_{V_2\to W} E^{V_2}_{t,j,a} + \left(1-\omega\right) E^W_{t,j,a}\right]$$
$$+ \sum_{j=1}^{n} m_{j,i,a-1}\, \delta_{a-1}\left(1-\mu_{a-1}\right)\left[p_{V_2\to W} E^{V_2}_{t,j,a-1} + \left(1-\omega\right) E^W_{t,j,a-1}\right], \quad \text{(S35)}$$

$$R^W_{t+1,i,a} = \sum_{j=1}^{n} m_{j,i,a} \left(1-\delta_a\right)\left(1-\mu_a\right)\left[p_{V_2\to W}\left(I^{V_2}_{t,j,a} + R^{V_2}_{t,j,a}\right) + \left(1-\omega\right)\left(I^W_{t,j,a} + R^W_{t,j,a}\right)\right]$$
$$+ \sum_{j=1}^{n} m_{j,i,a-1}\, \delta_{a-1}\left(1-\mu_{a-1}\right)\left[p_{V_2\to W}\left(I^{V_2}_{t,j,a-1} + R^{V_2}_{t,j,a-1}\right) \quad \text{(S36)}$$
$$+ \left(1-\omega\right)\left(I^W_{t,j,a-1} + R^W_{t,j,a-1}\right)\right].$$

At time $t = 0$, the livestock population on each island i was assumed to be entirely unprotected from any vaccine, and so for $a \in \{1, \ldots, A\}$ and $i \in \{1, \ldots, n\}$,

$$S^U_{0,i,a} = p_a \left[\left(1-\epsilon_i\right) N_i - E_{0,i} - I_{0,i}\right], \quad \text{(S37)}$$
$$E^U_{0,i,a} = p_a E_{0,i}, \quad \text{(S38)}$$
$$I^U_{0,i,a} = p_a I_{0,i}, \quad \text{(S39)}$$
$$R^U_{0,i,a} = \epsilon_i p_a N_i, \quad \text{(S40)}$$
$$\mathcal{X}^{\mathcal{V}}_{0,i,a} = 0, \qquad \text{for } \mathcal{X} \in \{S, E, I, R\} \text{ and } \mathcal{V} \in \{V_1, V_2, W\}, \quad \text{(S41)}$$

where N_i denoted the total livestock population on island i, $E_{0,i}$ denoted the number of exposed animals at time $t = 0$, $I_{0,i}$ denoted the number of exposed animals at time $t = 0$ and ϵ_i was the initial proportion of the population on each island i that have recovered from the virus.

Demographic and infection process parameterisation

The demographic and infection processes were parameterised as in the previous model by Tennant et al. [1]. Below, we describe the parameterisation of these processes in detail.

Using the Comoros archipelago as a case study to evaluate the impact of different vaccine strategies against RVFV, the number of islands in the metapopulation was set to four ($n = 4$). The livestock population was subdivided into 10 age groups ($A = 10$), with each age group representing livestock of 0–1 years old, 1–2 years old, ..., and greater than 9 years old. Only livestock in the first two age groups could be externally introduced ($A^{\text{ext}} = 2$) or moved between islands in the metapopulation ($A^{\text{move}} = 2$). The initial time ($t = 0$) corresponded to July 2004.

The initial number of exposed and infectious individuals was assumed to be small with $E_{0,i} = I_{0,i} = 5$. The population size of each island in the Comoros archipelago, N_i, was calculated using the Gridded Livestock Map of the World [2], with initial age distribution, p_a, set to the age profiles calculated by

Janelle et al. [3] and Tillard et al. [4]. Each time step in the model represented 1.08 weeks (an epidemiological week) as this allowed us to directly map four epidemiological weeks to a single calendar month of serological data during model fitting (see below). Thus the weekly ageing probability δ_a for the first nine age groups was set to 1/48 with $\delta_{10} = 0$. The mortality rates of age groups 1–9 and 10 were set to 8.8×10^{-3} and 6.2×10^{-3} respectively [3, 4]. Infectious imports were introduced into the system every 10 years ($t^{\text{ext}}_{(\text{freq})} = 480$).

The model in the absence of vaccination ($\psi = 0$) was fitted in a Bayesian framework to a series of monthly cross-sectional and annual longitudinal seroprevalence surveys conducted on each of the four islands in the Comoros archipelago from July 2004 until June 2015 ($t = 527$). Fitting the model to these data informed the remaining demographic and infection process parameters of the model: the probability of moving between islands per week, $m_{i,j}$, the island-specific disease transmission rate parameters, α and γ_i, external importation of livestock, I^{ext}_i, $t^{\text{ext}}_{(\text{start})}$ and $t^{\text{ext}}_{(\text{duration})}$, and initial proportion of the population immune to RVFV on each island, ϵ_i. S2 Table shows a list of posterior parameter estimates inferred through model fitting. Refer to Tennant et al. [1] for the full details and discussion of the model fitting procedure and results.

Optimisation of vaccine allocation

One goal of the study was to determine the optimal way to distribute vaccines across the archipelago. Given a number of vaccines to administer in the metapopulation per unit time, ψ as described above, vaccines were allocated to each island per unit time according to the vaccine distribution ρ. Here, we denote the complete set of possible vaccine allocations by P, where P defines an $(n - 1)$ simplex:

$$P = \left\{ \rho \in \mathbb{R}^n \;\middle|\; 0 \leq \rho_i \leq 1, \sum_{i \in \{1,\dots,n\}} \rho_i = 1 \right\}. \tag{S42}$$

We sought to optimise this distribution of vaccines by maximising some objective function f that depended on ρ. We denote the optimal vaccine distribution with ρ^*.

$$\rho^* = \arg\max_{\rho \in P} f(\rho). \tag{S43}$$

The objective function was defined in terms of the effectiveness of a given vaccine strategy, g as defined below, and included any uncertainty in model parameters, denoted by θ (refer to S2 Table for parameter values and ranges). Integrating over the joint distribution of model parameters $\pi(\theta)$ yields the following

objective function:

$$f(\rho) = \int_{\theta} g(\rho|\theta)\, \pi(\theta|y)\, d\theta, \tag{S44}$$

where $g(\rho|\theta)$ denotes the effectiveness of a given vaccine distribution ρ and model parameters θ, and $\pi(\theta|y)$ denotes the posterior distribution of the fitted model without vaccination as in [1].

Direct analysis of the above objective function is intractable, therefore we used Monte Carlo integration to approximate it. The following equation gives an unbiased estimate of the objective function for any given vaccine distribution ρ:

$$\hat{f}(\rho) = \frac{1}{K}\sum_{k=1}^{K} g(\rho|\theta_k), \qquad \theta_k \sim \pi(\theta|y), \tag{S45}$$

where θ_k denotes a single sample from the joint distribution of model parameters $\pi(\theta)$, K is the total number of random samples of model parameters, and $g(\rho|\theta_k)$ represents the effectiveness of a vaccine distribution ρ given a single sample of model parameters θ_k.

Optimisation algorithm

In order to determine the optimal allocation of vaccines, a sequential Monte Carlo (SMC) scheme was employed [5]. The optimisation algorithm worked by gradually concentrating a set of vaccine distributions, herein referred to as 'particles', into regions of parameter space associated with a larger evaluations of the objective function. The algorithm that we used consisted of three main components:

1. a sampling part, in which particles with a higher objective function evaluation were favoured,
2. a density-tempering part, which allowed particles to move from the initially proposed distribution of particles towards an empirical distribution of the objective function, and
3. a rejuvenation part, which meant that sampled particles would be jittered using a Metropolis-Hastings kernel with a (truncated) multivariate Gaussian proposal distribution.

Below we describe each of these three components. The complete optimisation algorithm and associated notation can be found in Algorithm S1.

Sampling particles

The algorithm aimed to obtain a set of vaccine distributions that were representative of the objective function $f(\rho)$. However, the objective function may not be positive for all $\rho \in P$ and may not di-

Input : $J \geq 1$, // Number of SMC particles
$K \geq 1$, // Number of effectiveness samples
$I(\rho)$, // Initial distribution of SMC particles
$g(\rho|\theta)$, // Effectiveness function
$\pi(\theta)$, // Joint distribution of model parameters
$m \geq 1$, // Scalar for objective function
$d_1 \in [0, 1]$, // First tempering weight of objective function
$q(.|\rho)$, // Proposal distribution
$c_{\max} > 0$. // Maximum cumulative acceptance rate

Output: ρ^* such that $f(\rho^*) = \max_{\rho \in P} f(\rho) = \max_{\rho \in P} \int_\theta g(\rho|\theta)\, h(\theta)\, \mathrm{d}\theta$.

// Initialisation of particles and initial evaluation of objective function.
for $j = 1$ **to** J **do**
  $w_j^{(1)} \leftarrow 1/J$;
  $\rho_j^{(1)} \sim I(\rho)$;
  for $k = 1$ **to** K **do**
    $\theta_k \sim \pi(\theta)$;
    $g_{jk} \leftarrow g\left(\rho_j^{(1)}|\theta_k\right)$;
  end
  $\hat{f}_j \leftarrow \frac{1}{K}\sum_{k=1}^{K} g_{jk}$;
  $h_j^{(1)} \leftarrow \exp\left(m\hat{f}_j\right)^{d_1} I(\rho_j)^{(1-d_1)}$;
end
$s \leftarrow 1$; // Progress through tempering weights until the initial distribution vanishes.
while $d_s < 1$ **do**
  Find the largest $d_{s+1} \in [d_s, 1]$ such that $\left(\sum_{j=1}^{J} w_j^{(s+1)}\right)^2 / \sum_{j=1}^{J}\left(w_j^{(s+1)}\right)^2 \geq J/2$ with
    $w_j^{(s+1)} = w_j^{(s)} h_j^{(s+1)}/h_j^{(s)}$ and $h_j^{(s)} = \exp\left(m\hat{f}_j\right)^{d_s} I(\rho_j)^{(1-d_s)}$;
  Sample $\rho_j^{(s+1)}$ from $\rho_j^{(s)}$ with weights $w_j^{(s+1)}$;
  $c \leftarrow 0$; // Jitter particles using a Metropolis-Hastings algorithm.
  while $c < c_{\max}$ **do**
    for $j = 1$ **to** J **do**
      $\rho'_j \sim q(.|\rho_j)$;
      for $k = 1$ **to** K **do**
        $\theta_k \sim \pi(\theta)$;
        $g'_{jk} \leftarrow g\left(\rho'_j|\theta_k\right)$;
      end
      $\hat{f}_j{}' \leftarrow \frac{1}{K}\sum_{k=1}^{K} g_{jk}$;
      $h'_j \leftarrow \exp\left(m\hat{f}_j{}'\right)^{d_{s+1}} I\left(\rho'_j\right)^{(1-d_{s+1})}$;
      $\alpha \leftarrow \left[h'_j q\left(\rho'_j|\rho_j\right)\right] / \left[h_j^{(s+1)} q\left(\rho_j|\rho'_j\right)\right]$;
      $v \sim U(0, 1)$;
      if $\alpha < \min\{1, v\}$ **then**
        $\rho_j^{(s+1)} \leftarrow \rho'_j$;
        $h_j^{(s+1)} \leftarrow h'_j$;
        $c \leftarrow c + 1/J$;
      end
    end
  end
  $s \leftarrow s + 1$;
end
$j^* \leftarrow \arg\max_{1 \leq j \leq J} h_j^{(s)}$; // Extract the particle with the largest evaluation.
$\rho^* \leftarrow \rho_{j^*}$;

Algorithm S1: Sequential Monte Carlo (SMC) optimisation algorithm. Pseudo-code of the algorithm used to determine the optimal distribution of vaccines across all islands in the Comoros archipelago. Here, particles corresponded to a unique distribution of vaccines, and the objective function corresponded to the mean effectiveness of a given strategy. The algorithm included tempering from the initial distribution of particles towards the distribution of particles governed by the objective function.

rectly define a probability density function. Instead, we obtained samples from the following monotonic transformation of the objective function:

$$h(\rho) \propto \exp\left[m f(\rho)\right], \tag{S46}$$

where $m \geq 1$ defines the peakiness of the transformed objective function.

The transformed function defined a probability density function (up to an unknown normalising constant) from which particles could be sampled. As the transformation was (positive) monotonic, the optimal vaccine distribution for $h(\rho)$ was the same as the objective function $f(\rho)$:

$$\rho_j \sim h(.), \qquad j \in \{1, ..., J\}, \tag{S47}$$

$$\hat{\rho}^* = \arg\max_{j\in\{1,\dots,J\}} h(\rho_j) = \arg\max_{j\in\{1,\dots,J\}} f(\rho_k), \tag{S48}$$

where ρ_j denotes a single sample from the transformed objective function, J is the total number of particles to be sampled, and $\hat{\rho}^*$ is an estimate of the optimal vaccine distribution.

In order to obtain samples from the transformed objective function, we first sampled particles from some known sampling distribution, denoted $I(\rho)$, and used importance sampling to filter the particles towards $h(\rho)$. To filter the initial set of particles in a single step may lead to a poorly representative sample of $h(\rho)$, so we introduced an intermediate set of density-tempered distributions, denoted $h_{d_s}(\rho)$, which allowed a more smooth transition from $I(\rho)$ to $h(\rho)$ across multiple steps.

Intermediate density-tempered distributions

At each step s in the filtering process, we defined the intermediate density-tempered distributions as:

$$h_{d_s}(\rho) = h(\rho)^{d_s} I(\rho)^{(1-d_s)}, \tag{S49}$$

where $d_s \in [0, 1]$ is the density-tempering parameter at step s, which monotonically increase with s. By setting $d_1 = 0$, the first intermediate density-tempered distribution, $h_{d_1}(\rho)$, is equivalent to the initial distribution $I(\rho)$.

Given a set of particles representing $h_{d_s}(\rho)$, the particles were filtered to the next intermediate distribution $h_{d_{s+1}}(\rho)$ by first calculating the following importance weights:

$$w_j^{(s+1)} = w_j^{(s)} \frac{h_{d_{s+1}}\left(\rho_j^{(s)}\right)}{h_{d_s}\left(\rho_j^{(s)}\right)}, \tag{S50}$$

$$w_j^{(1)} := \frac{1}{J}, \tag{S51}$$

where $w_j^{(s)}$ denotes the importance weight at step s, and $\rho_j^{(s)}$ is a sample from the intermediate density-tempered distribution $h_{d_s}(\rho)$. The importance weights were then used to sample the next set of particles $\rho_j^{(s+1)}$. Using this approach, the normalising constant of $h(\rho)$ did not need to be known.

To ensure a smooth transition between intermediate density-tempered distributions, the sequence of density-tempering parameters was chosen to be the largest $d_{s+1} \in [d_s, 1]$ such that the effective sample size (ESS) from one intermediate distribution to the next remained above $J/2$, where

$$\mathrm{ESS} = \frac{\left(\sum_{j=1}^{J} w_j^{(s+1)}\right)^2}{\sum_{j=1}^{J}\left(w_j^{(s+1)}\right)^2}. \tag{S52}$$

Particle rejuvenation

Repeatedly filtering particles from one intermediate distribution to the next alone would result in a lack of diversity among particles, leading to a poor representation of the final distribution $h(\rho)$. To ameliorate this, we used a Metropolis-Hastings step to jitter and rejuvenate the set of particles.

After each resampling step in the particle filter, we proposed a new particle ρ_j' from a proposal distribution $q(.|\rho_j)$ for all $j \in \{1, ..., J\}$. The proposed particle ρ_j' was then evaluated using the current intermediate distribution, and replaced the previous particle ρ_j with probability

$$\alpha = \min\left\{1, \frac{h\left(\rho_j'\right)^{d_s} I\left(\rho_j'\right)^{1-d_s}}{h\left(\rho_j\right)^{d_s} I\left(\rho_j\right)^{1-d_s}} \frac{q\left(\rho_j|\rho_j'\right)}{q\left(\rho_j'|\rho_j\right)}\right\}, \tag{S53}$$

where $h\left(\rho_j'\right)^{d_s} I\left(\rho_j'\right)^{1-d_s}$ denotes the intermediate distribution at step s. This procedure was repeated until a cumulative acceptance rate, $c_{\max}$, was reached.

In this work the proposal distribution $q(.|\rho_j)$ was selected to be a mixture distribution with equal weights between: (i) the initialisation distribution I, (ii) a multivariate Gaussian with mean ρ_j and covariance equal to the identity matrix scaled by $\frac{0.1^2}{n}$, representing a small random walk around the current particle ρ_j, and (iii) a multivariate Gaussian with mean and covariance matrix calculated directly from the entire set of particles ρ. This proposal distribution allowed the parameter space to be continuously explored, whilst considering information from the current set of particles [6].

Optimal particle

Particles were filtered sequentially through each intermediate distribution and rejuvenated in turn up to step S with $d_S = 1$. This yielded a set of particles representing the transformed objective function $h(\rho)$. An estimate for the optimal vaccine distribution was determined as the vaccine distribution ρ which

maximised $h(\rho)$ as in Equation S49.

References

[1] Tennant, W. S., Cardinale, E., Cêtre-Sossah, C., Moutroifi, Y., Le Godais, G., Colombi, D., Spencer, S. E., Tildesley, M. J., Keeling, M. J., Charafouddine, O., et al. (2021). Modelling the persistence and control of Rift Valley fever virus in a spatially heterogeneous landscape. *Nature communications*, 12(1):5593.

[2] Gilbert, M., Nicolas, G., Cinardi, G., Van Boeckel, T. P., Vanwambeke, S. O., Wint, G., and Robinson, T. P. (2018). Global distribution data for cattle, buffaloes, horses, sheep, goats, pigs, chickens and ducks in 2010. *Scientific data*, 5(1):1–11.

[3] Janelle, J., Issoufi, A., Grimaldine, A., and Tillard, E. (2013). Référentiel technico-économique des élevages d'ovins et de caprins à Mayotte. *Centre de cooperation internationale en recherche agronomique pour le developpement*, 1.

[4] Tillard, E., Moussa, T., Balberini, L., Aubriot, D., and Berre, D. (2013). Référentiel technico-économique des élevages de bovins à mayotte. *Centre de cooperation internationale en recherche agronomique pour le developpement*, 1.

[5] Duan, J., Li, S., and Xu, Y. (2023). Sequential Monte Carlo optimization and statistical inference. *Wiley Interdisciplinary Reviews: Computational Statistics*, 15(3):e1598.

[6] Chopin, N. (2002). A sequential particle filter method for static models. *Biometrika*, 89(3):539–552.
