# Supplementary material for: Effectiveness and equity of vaccination strategies against Rift Valley fever in a heterogeneous landscape
Source: PLoS Negl Trop Dis. 2025 Jul 28;19(7):e0013346. doi: 10.1371/journal.pntd.0013346 (PMC12316399; doi:10.1371/journal.pntd.0013346)
Supplement: S1 Table — A mathematical model describing livestock infection with and vaccination against Rift Valley Fever virus across the four islands in the Comoros archipelago was developed. In order to determine how best to distribute a set number of vaccines across the archipelago, a Sequential Monte Carlo optimisation algorithm was used, where the objective function was the number of infections averted between July 2015 and June 2050. The table shows all mathematical notation used to describe the model. (PDF) [file pntd.0013346.s003.pdf]

S1 Table. Mathematical notation for livestock infection model. A mathematical model describing livestock infection with and vaccination against Rift Valley Fever virus across the four islands in the Comoros archipelago was developed. In order to determine how best to distribute a set number of vaccines across the archipelago, a Sequential Monte Carlo optimisation algorithm was used, where the objective function was the number of infections averted between July 2015 and June 2050. The table shows all mathematical notation used to describe the model.

Notation	Description
S	Number of susceptible livestock
E	Exposed (infected, but not yet infectious) livestock
I	Infectious livestock
R	Recovered (with life-long natural immunity) livestock
U	Unvaccinated livestock
V_1	Vaccinated livestock which are not yet protected (stage 1)
V_2	Vaccinated livestock which are not yet protected (stage 2)
W	Vaccinated and protected livestock
$\mathcal{X}^{\mathcal{V}}$	Number of livestock with status $\mathcal{X} \in \{S, E, I, R\}$ and $\mathcal{V} \in \{U, V_1, V_2, W\}$
I^{ext}	Externally introduced infectious unvaccinated livestock
t	Time (epidemiological weeks)
i	Island
a	Age group
n	Number of islands in the metapopulation
A	Number of age groups
A^V	Maximum age group that can be vaccinated
A^{move}	Maximum age group that could be moved between islands
A^{ext}	Maximum age group of livestock that are externally introduced
$t^{\text{ext}}_{(\text{start})}$	Start of infectious imports
$t^{\text{ext}}_{(\text{duration})}$	Duration of infectious imports
$t^{\text{ext}}_{(\text{freq})}$	Frequency of infectious import events
t_V	Time that vaccination begins
T	Maximum simulation time
p_a	Initial proportion of livestock in age group a
ϵ_i	Initial proportion of livestock immune on island i
$\nu_{t,i}$	Number of births at time t on island i
μ_a	Probability of dying in age group a per week
δ_a	Probability of ageing out from age group a per week
$m_{j,i,a}$	Probability of livestock from age group a moving from island j to island i per week
$\lambda_{t,i}$	Probability of becoming infected on island i at time t
$\beta_{t,i}$	Disease transmission rate per week
α	Influence of Normalised Difference Vegetation Index on the disease transmission rate
γ_i	Natural log of the minimum disease transmission rate on island i per week
p^{eff}	Vaccine efficacy
$p_{V_1 \to V_2}$	Proportion of livestock with vaccination status V_1 moving to V_2 per week
$p_{V_2 \to W}$	Proportion of livestock with vaccination status V_2 moving to W per week
τ_ω	Mean duration of vaccine-induced immunity (weeks)
ω	Probability of vaccine-induced immunity waning per week
$\xi_{t,i,a}$	Proportion of individuals from age group a and island i at time t being vaccinated
ψ	Number of vaccine doses to administer across the metapopulation per week
ρ_i	Proportion of vaccines allocated to island i
