# Supplementary material for: Effectiveness and equity of vaccination strategies against Rift Valley fever in a heterogeneous landscape
Source: PLoS Negl Trop Dis. 2025 Jul 28;19(7):e0013346. doi: 10.1371/journal.pntd.0013346 (PMC12316399; doi:10.1371/journal.pntd.0013346)
Supplement: S2 Table — Model parameters were set to evaluate the impact of different vaccine strategies against Rift Valley fever virus in the Comoros archipelago. The table shows the values of model parameters used in simulating the transmission model forward in time. Some demographic parameters were fixed and chosen to reflect the livestock demography of the Comoros archipelago, and all vaccine parameters were chosen based on the World Health Organization target product profiles for vaccines and field studies on existing vaccines in livestock. The remaining parameters were estimated by fitting the model without vaccination to serological data in a Bayesian framework and are represented by the median and 95% credible interval of 10,000 posterior samples in the table. Full justification of vaccine parameters is described in the main text, with the justification for parameters for the demographic and infection processes described in S1 Text. (PDF) [file pntd.0013346.s004.pdf]

**S2 Table. Summary of parameters used during model simulations.** Model parameters were set to evaluate the impact of different vaccine strategies against Rift Valley fever virus in the Comoros archipelago. The table shows the values of model parameters used in simulating the transmission model forward in time. Some demographic parameters were fixed and chosen to reflect the livestock demography of the Comoros archipelago, and all vaccine parameters were chosen based on the World Health Organization target product profiles for vaccines and field studies on existing vaccines in livestock. The remaining parameters were estimated by fitting the model without vaccination to serological data in a Bayesian framework and are represented by the median and 95% credible interval of 10,000 posterior samples in the table. Full justification of vaccine parameters is described in the main text, with the justification for parameters for the demographic and infection processes described in S1 Text.

| Parameter | Description | Value |
|---|---|---|
| $n$ | Number of islands in the metapopulation | 4 |
| $A$ | Number of age groups | 10 |
| $A^{\text{move}}$ | Maximum age group moved between islands | 2 |
| $A^{\text{ext}}$ | Maximum age group of imported livestock | 2 |
| $t^{\text{ext}}_{\text{(freq)}}$ | Frequency of infectious import events | 480 |
| $t_V$ | Time that vaccination begins | 528 |
| $T$ | Maximum simulation time | 2207 |
| $\mu_a$ | Probability of dying per week ($a \in \{1, ..., 9\}$) | 0.0088 |
| $\mu_{10}$ | Probability of dying in age group 10 per week | 0.0062 |
| $\delta_a$ | Probability of ageing per week ($a \in \{1, ..., 9\}$) | 1/48 |
| $p^{\text{eff}}$ | Vaccine efficacy | 0.9 |
| $p_{V_1 \to V_2}$ | Proportion of livestock in $V_1$ moving to $V_2$ per week | 1 |
| $p_{V_2 \to W}$ | Proportion of livestock in $V_2$ moving to $W$ per week | 1 |
| $\tau_\omega$ | Mean duration of vaccine-induced immunity (weeks) | 96 |

| Parameter | Description | Values |
|---|---|---|
| $A^V$ | Maximum age group that can be vaccinated | $\{2, 10\}$ |
| $\psi / \sum_{i=1}^{4} N_i$ | Proportion of livestock vaccinated per week | $\{0.05, 0.1, 0.15, 0.2, 0.25, 0.3\}$ |

| Parameter | Description | Median [95% credible interval] |
|---|---|---|
| $48N_1m_{1,2}$ | Annual movement from Grande Comore to Mohéli | 329.33 [266.54, 391.15] |
| $48N_1m_{1,3}$ | Annual movement from Grande Comore to Anjouan | 65.11 [7.82, 137.33] |
| $48N_2m_{2,1}$ | Annual movement from Mohéli to Grande Comore | 418.27 [321.79, 517.59] |
| $48N_2m_{2,3}$ | Annual movement from Mohéli to Anjouan | 73.44 [18.43, 139.73] |
| $48N_3m_{3,1}$ | Annual movement from Anjouan to Grande Comore | 624.58 [515.76, 735.8] |
| $48N_3m_{3,2}$ | Annual movement from Anjouan to Mohéli | 407.93 [339.02, 476.64] |
| $48N_3m_{3,4}$ | Annual movement from Anjouan to Mayotte | 1896.24 [1653.36, 2126.16] |
| $\epsilon_1$ | Proportion immune at time $t = 0$ in Grande Comore | 0.372 [0.284, 0.429] |
| $\epsilon_2$ | Proportion immune at time $t = 0$ in Mohéli | 0.397 [0.346, 0.462] |
| $\epsilon_3$ | Proportion immune at time $t = 0$ in Anjouan | 0.035 [0.015, 0.063] |
| $\epsilon_4$ | Proportion immune at time $t = 0$ in Mayotte | 0.141 [0.107, 0.178] |
| $\alpha$ | Seasonal transmission component scalar | 7.73 [7.09, 8.42] |
| $\gamma_1$ | Transmission constant for Grande Comore | -0.79 [-0.89, -0.69] |
| $\gamma_2$ | Transmission constant for Mohéli | -0.79 [-0.91, -0.67] |
| $\gamma_3$ | Transmission constant for Anjouan | -1.06 [-1.18, -0.96] |
| $\gamma_4$ | Transmission constant for Mayotte | -1.02 [-1.14, -0.92] |
| $t^{\text{ext}}_{\text{(start)}}$ | Start of imports into Grande Comore | 130.69 [116.94, 151.70] |
| $t^{\text{ext}}_{\text{(duration)}}$ | Duration of imports into Grande Comore | 23.13 [5.15, 40.68] |
| $48I^{\text{ext}}_1$ | Annual infectious imports into Grande Comore | 175.34 [13.82, 414.82] |
