# Supplementary material for: Effectiveness and equity of vaccination strategies against Rift Valley fever in a heterogeneous landscape
Source: PLoS Negl Trop Dis. 2025 Jul 28;19(7):e0013346. doi: 10.1371/journal.pntd.0013346 (PMC12316399; doi:10.1371/journal.pntd.0013346)
Supplement: S1 Fig — Vaccines were allocated to each of the four islands in the archipelago either proportionally to the livestock population size of each island (grey dashed line), optimally to maximise the percentage of infections averted across the archipelago (orange violins), or the percentage of infections averted on the island with the the worst performance (blue violins). For both optimal vaccine allocations, all vaccination rates and livestock tagging strategies, the median percentage of livestock vaccinated on Mohéli was greater than the overall percentage of livestock vaccinated across the archipelago, indicating overall favour towards vaccinating Mohéli. The violins show the percentage of animals vaccinated annually on each island for different annual vaccination rates, allocation methods and tagging strategies. The points and boxplots show the median and inter-quartile range for each scenario respectively. All violins shown are based on 500 executions of the optimisation algorithm. (PDF) [file pntd.0013346.s005.pdf]

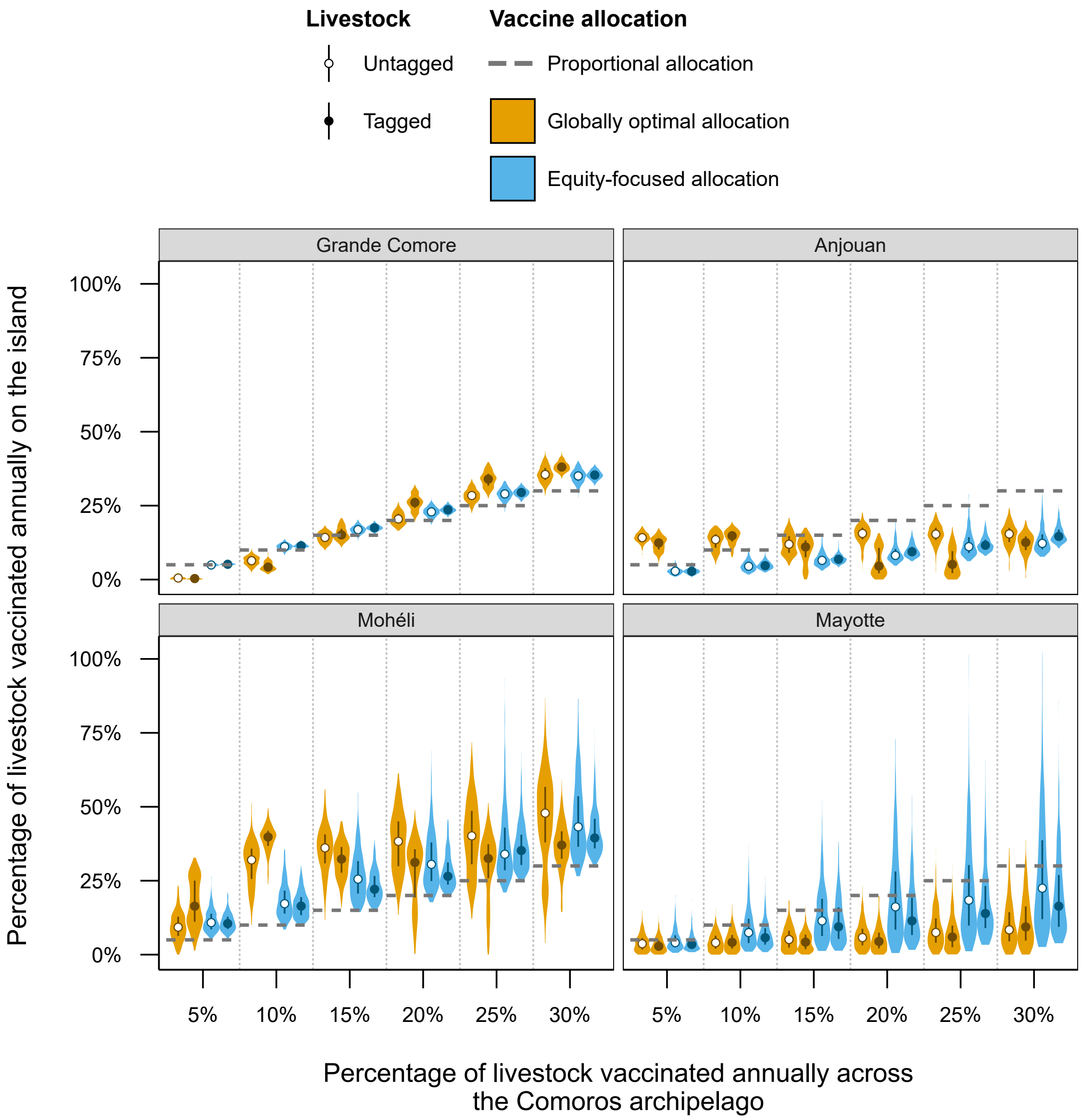

S1 Fig. Animals vaccinated on each island in the Comoros archipelago for different vaccine strategies. Vaccines were allocated to each of the four islands in the archipelago either proportionally to the livestock population size of each island (grey dashed line), optimally to maximise the percentage of infections averted across the archipelago (orange violins), or the percentage of infections averted on the island with the the worst performance (blue violins). For both optimal vaccine allocations, all vaccination rates and livestock tagging strategies, the median percentage of livestock vaccinated on Mohéli was greater than the overall percentage of livestock vaccinated across the archipelago, indicating overall favour towards vaccinating Mohéli. The violins show the percentage of animals vaccinated annually on each island for different annual vaccination rates, allocation methods and tagging strategies. The points and boxplots show the median and inter-quartile range for each scenario respectively. All violins shown are based on 500 executions of the optimisation algorithm.
