# Supplementary material for: Effectiveness and equity of vaccination strategies against Rift Valley fever in a heterogeneous landscape
Source: PLoS Negl Trop Dis. 2025 Jul 28;19(7):e0013346. doi: 10.1371/journal.pntd.0013346 (PMC12316399; doi:10.1371/journal.pntd.0013346)
Supplement: S3 Fig — Shown is the weekly model predicted median (solid line) and 95% (shaded area) prediction interval of the percentage of vaccines that were administered to livestock without vaccine-induced nor natural protection against Rift Valley fever virus on each island in the Comoros archipelago—Grande Comore (red), Mohéli (blue), Anjouan (green) and Mayotte (purple)—when vaccinating 10% and 15% of livestock across the archipelago annually under both optimal vaccine allocations and tagging strategies. Summary metrics were generated using 1,000 model simulations. (PDF) [file pntd.0013346.s007.pdf]

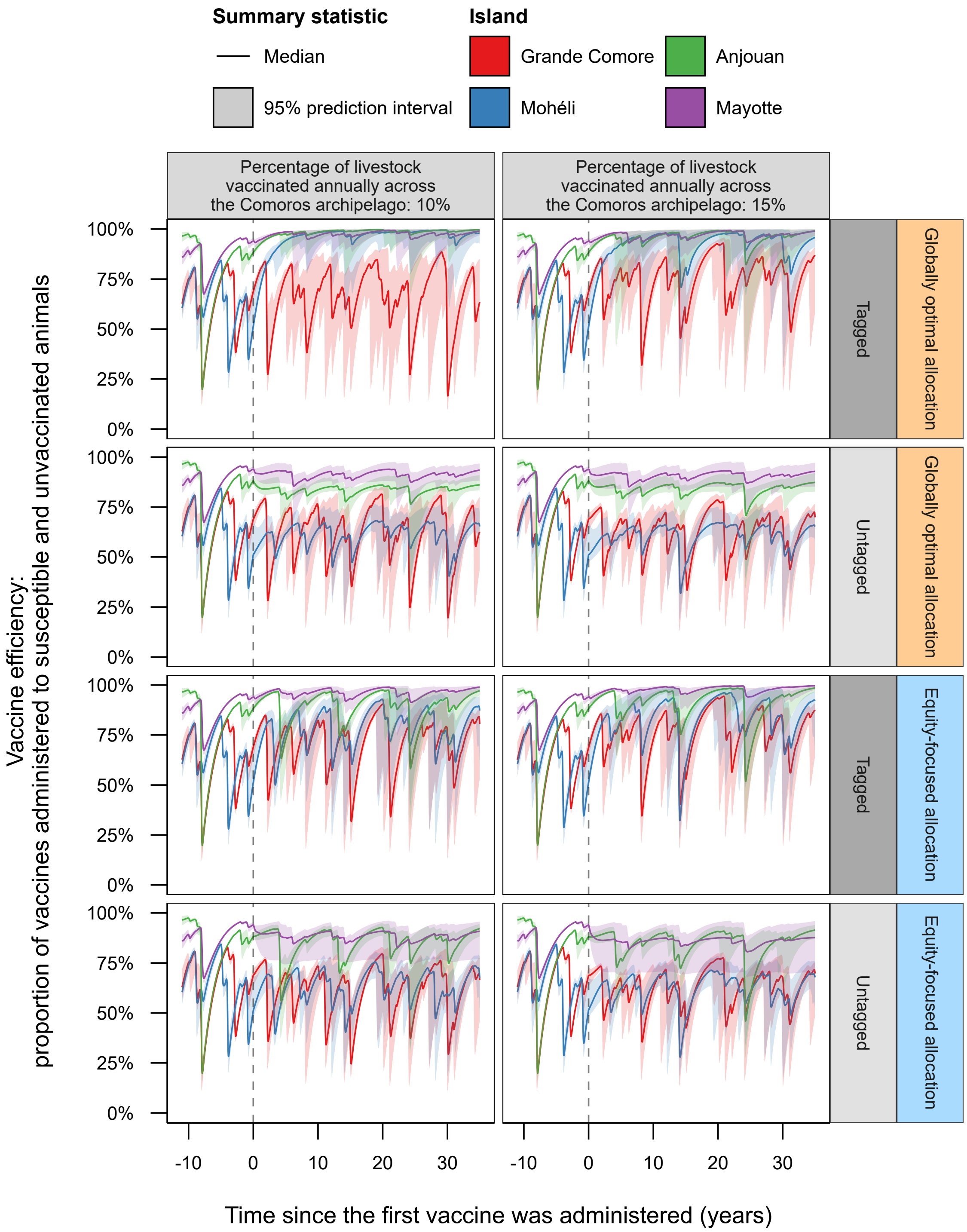

S3 Fig. Efficiency of vaccine administration on each island in the Comoros archipelago at 10% and 15% vaccination rates.. Shown is the weekly model predicted median (solid line) and 95% (shaded area) prediction interval of the percentage of vaccines that were administered to livestock without vaccine-induced nor natural protection against Rift Valley fever virus on each island in the Comoros archipelago—Grande Comore (red), Mohéli (blue), Anjouan (green) and Mayotte (purple)—when vaccinating 10% and 15% of livestock across the archipelago annually under both optimal vaccine allocations and tagging strategies. Summary metrics were generated using 1,000 model simulations.
