# Supplementary material for: Effectiveness and equity of vaccination strategies against Rift Valley fever in a heterogeneous landscape
Source: PLoS Negl Trop Dis. 2025 Jul 28;19(7):e0013346. doi: 10.1371/journal.pntd.0013346 (PMC12316399; doi:10.1371/journal.pntd.0013346)
Supplement: S5 Fig — Vaccines were allocated to each of the four islands in the archipelago either proportionally to the livestock population size of each island (grey dashed line), optimally to maximise the percentage of infections averted across the archipelago when vaccinating all livestock (orange violins) or targeting only young livestock (green violins). The violins show the percentage of animals vaccinated annually on each island for different annual vaccination rates, allocation methods and tagging strategies. The points and boxplots show the median and inter-quartile range for each scenario respectively. All violins shown are based on 500 executions of the optimisation algorithm. (PDF) [file pntd.0013346.s009.pdf]

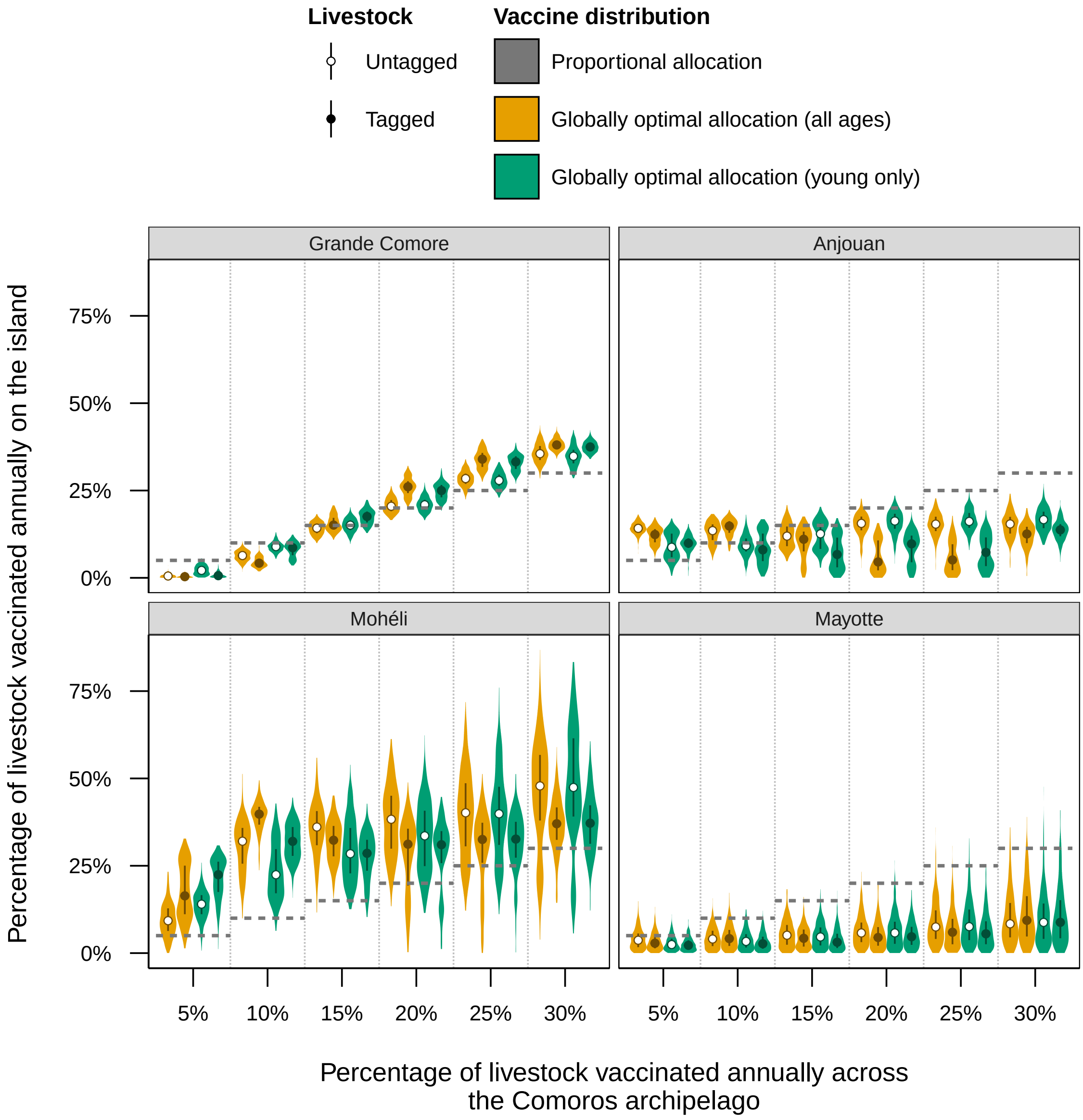

S5 Fig. Animals vaccinated on each island in the Comoros archipelago when targeting vaccine efforts to young livestock. Vaccines were allocated to each of the four islands in the archipelago either proportionally to the livestock population size of each island (grey dashed line), optimally to maximise the percentage of infections averted across the archipelago when vaccinating all livestock (orange violins) or targetting only young livestock (green violins). The violins show the percentage of animals vaccinated annually on each island for different annual vaccination rates, allocation methods and tagging strategies. The points and boxplots show the median and inter-quartile range for each scenario respectively. All violins shown are based on 500 executions of the optimisation algorithm.
