# Supplementary material for: Effectiveness and equity of vaccination strategies against Rift Valley fever in a heterogeneous landscape
Source: PLoS Negl Trop Dis. 2025 Jul 28;19(7):e0013346. doi: 10.1371/journal.pntd.0013346 (PMC12316399; doi:10.1371/journal.pntd.0013346)
Supplement: S6 Fig — The allocation of vaccines were optimised for each vaccination rate and tagging strategy assuming that vaccines were administered across all age groups and throughout the epidemiological year. Using these vaccine allocations, the effectiveness of only vaccinating within the first epidemiological month (July) or throughout the epidemiological year were simulated using the model. Shown is the median and 95% prediction interval of the percentage of infections averted across the Comoros archipelago for each scenario. All metrics shown were based on 25,000 model simulations. (PDF) [file pntd.0013346.s010.pdf]

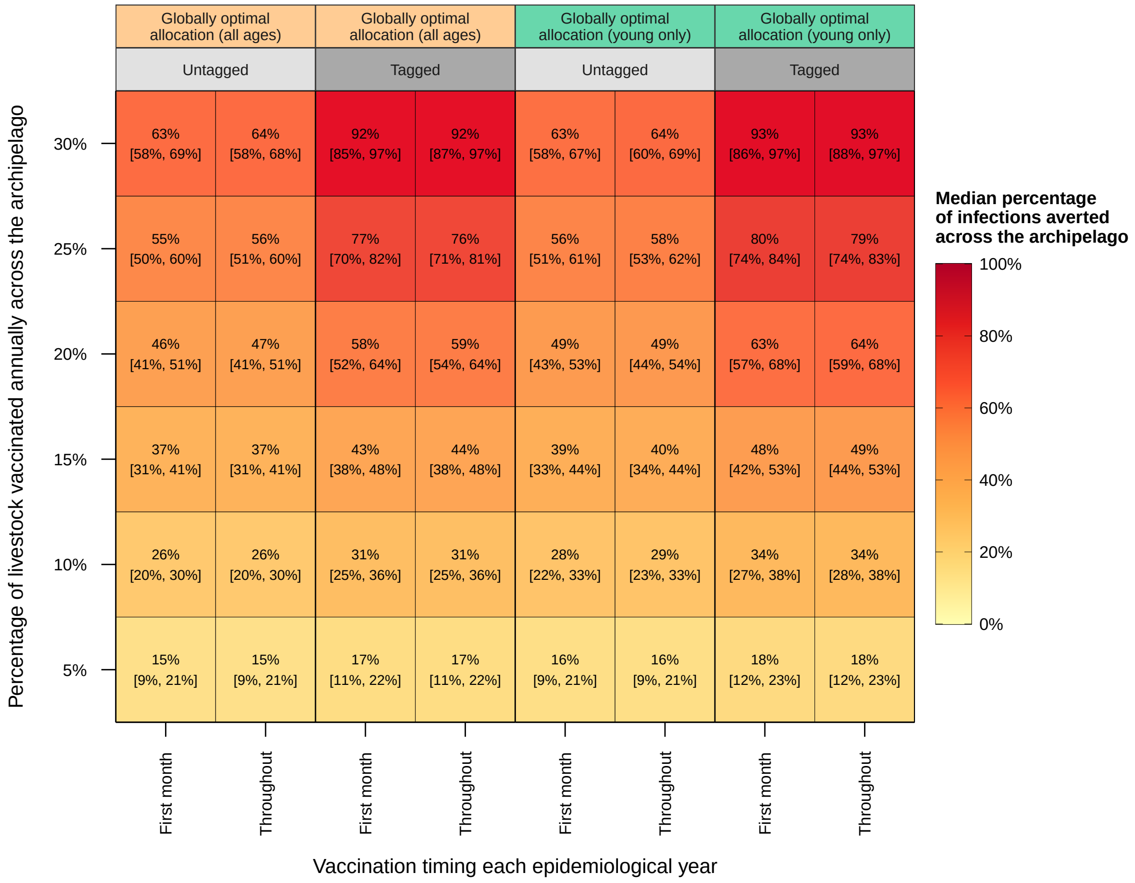

S6 Fig. Effectiveness of targeting vaccine efforts to young livestock and only administering vaccines in the first epidemiological month of the year. The allocation of vaccines were optimised for each vaccination rate and tagging strategy assuming that vaccines were administered throughout the epidemiological year when vaccinating all age groups and only young livestock. Using these vaccine allocations, the effectiveness of only vaccinating the first two age groups and / or within the first epidemiological month (July) were simulated using the model. Shown is the median and 95% prediction interval of the percentage of infections averted across the Comoros archipelago for each scenario. All metrics shown were based on 25,000 model simulations.
