# Supplementary material for: Effectiveness and equity of vaccination strategies against Rift Valley fever in a heterogeneous landscape
Source: PLoS Negl Trop Dis. 2025 Jul 28;19(7):e0013346. doi: 10.1371/journal.pntd.0013346 (PMC12316399; doi:10.1371/journal.pntd.0013346)
Supplement: S7 Fig — The allocation of vaccines were optimised for each vaccination rate and tagging strategy assuming that vaccines were administered across all age groups and throughout the epidemiological year. Using these optimal vaccine allocation, the effectiveness of only vaccinating every two or three years, alongside only vaccinating within the first epidemiological month (July), were simulated using the model for a reduced set of vaccination rates. Shown is the median and 95% prediction interval of the percentage of infections averted across the Comoros archipelago for each scenario. All metrics shown were based on 25,000 model simulations. (PDF) [file pntd.0013346.s011.pdf]

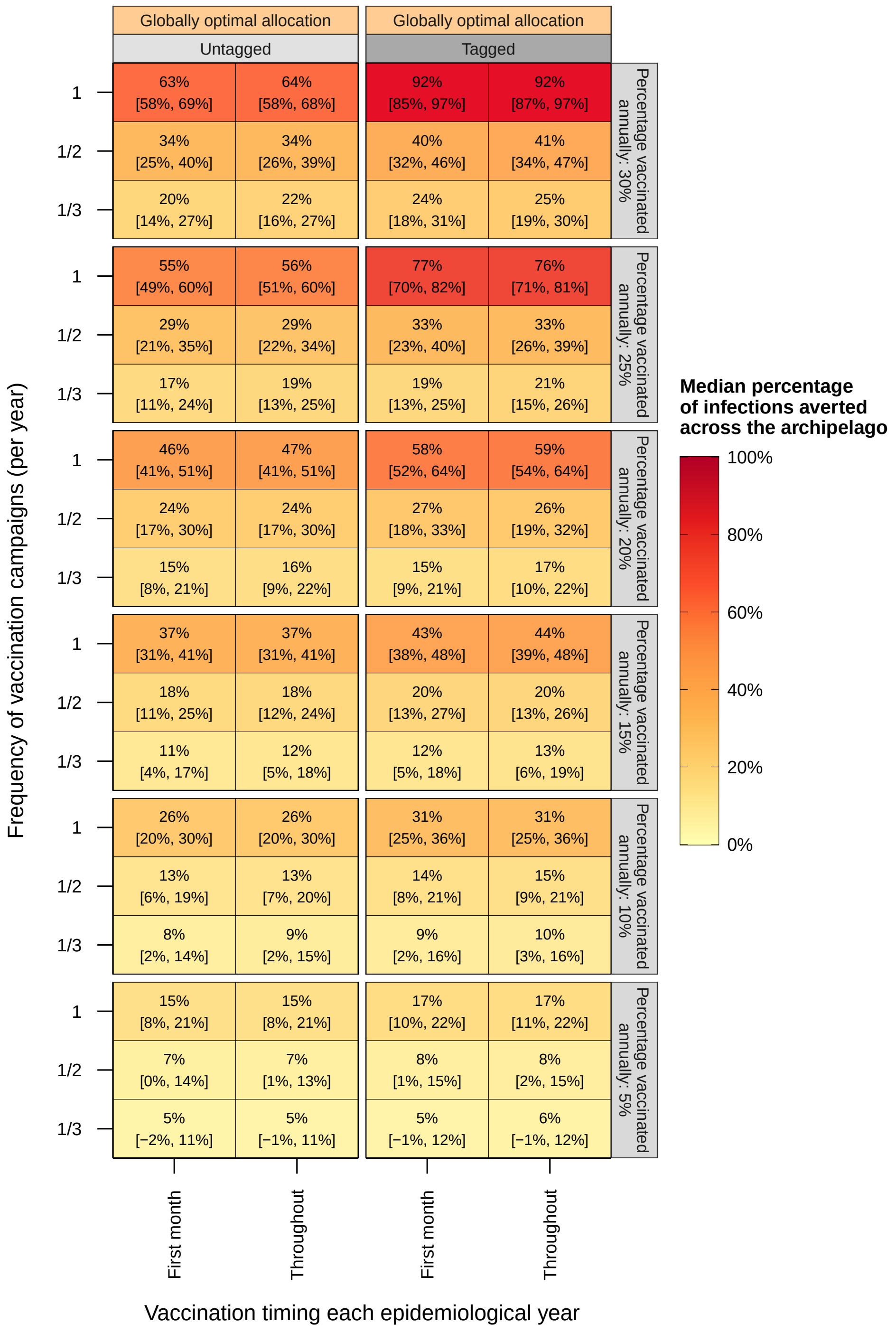

S7 Fig. Effectiveness with altered timing and frequency of vaccine administration. The allocation of vaccines were optimised for each vaccination rate and tagging strategy assuming that vaccines were administered across all age groups and throughout the epidemiological year. Using these optimal vaccine allocation, the effectiveness of only vaccinating every two or three years, alongside only vaccinating within the first epidemiological month (July), were simulated using the model for a reduced set of vaccination rates. Shown is the median and 95% prediction interval of the percentage of infections averted across the Comoros archipelago for each scenario. All metrics shown were based on 25,000 model simulations.
