# Supplementary material for: Effectiveness and equity of vaccination strategies against Rift Valley fever in a heterogeneous landscape
Source: PLoS Negl Trop Dis. 2025 Jul 28;19(7):e0013346. doi: 10.1371/journal.pntd.0013346 (PMC12316399; doi:10.1371/journal.pntd.0013346)
Supplement: S8 Fig — For six annual vaccination rates, vaccines were allocated to islands in the Comoros archipelago either proportionally to the population size of each island (grey violins), optimally in terms of infections averted across the archipelago (orange violins) or optimally in terms of infections averted on the worst-performing island (blue). Livestock were also either tagged post-vaccination (black circles) or not (white circles). For the majority of vaccination rates, vaccine allocations and tagging strategies, Grande Comore averted the lowest percentage of infections on average (grey boxes), and tagging livestock resulted in a greater number of infections averted across the archipelago. The violins show the percentage of infections averted across on each island for different annual vaccination rates, allocation methods and tagging strategies. The points and boxplots show the median and inter-quartile range for each scenario respectively. All metrics shown were based on 25,000 model simulations. (PDF) [file pntd.0013346.s012.pdf]

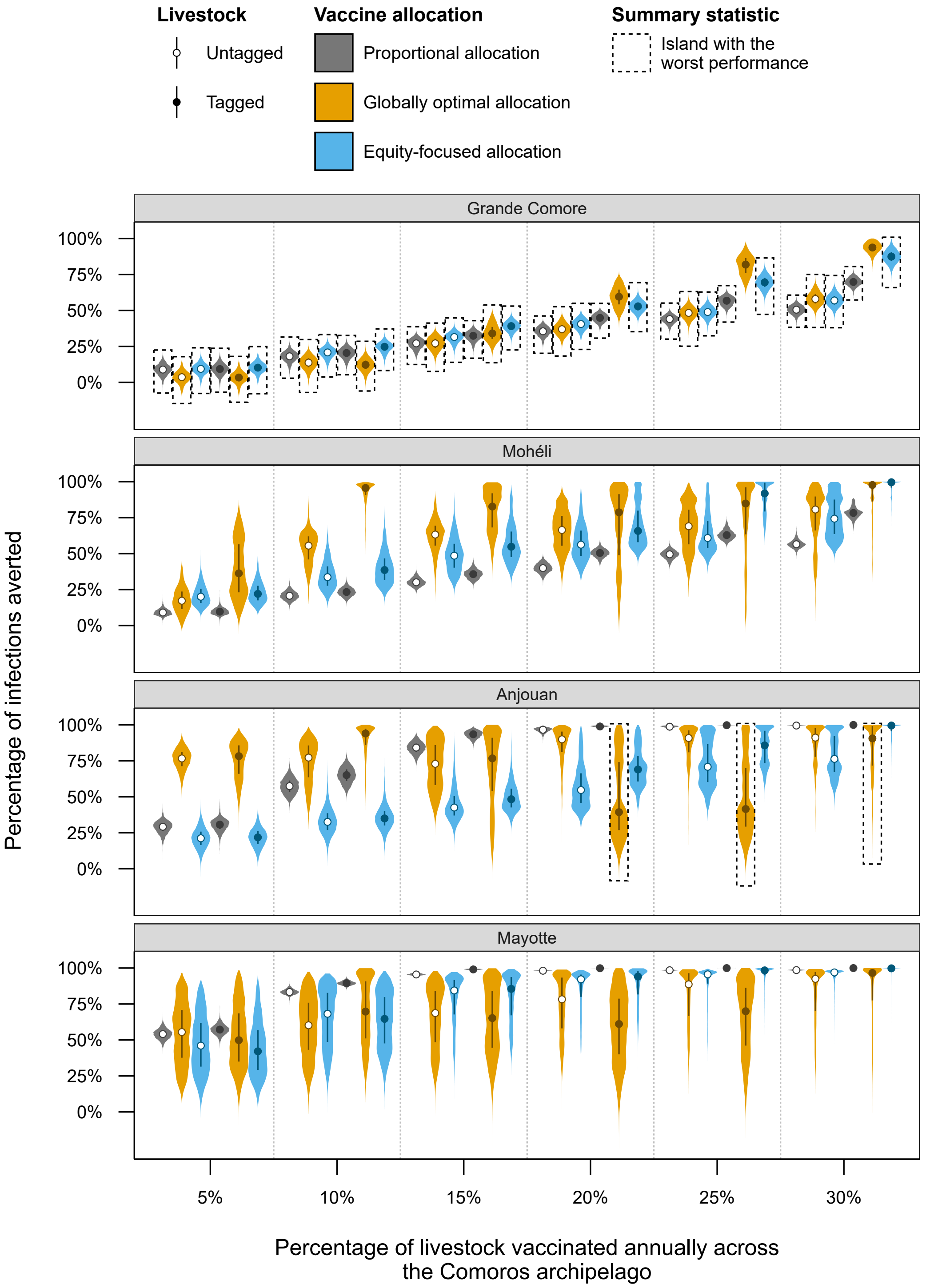

S8 Fig. Effectiveness of different vaccine strategies against Rift Valley fever virus on each island in the Comoros archipelago. For six annual vaccination rates, vaccines were allocated to islands in the Comoros archipelago either proportionally to the population size of each island (grey violins), optimally in terms of infections averted across the archipelago (orange violins) or optimmaly in terms of infections averted on the worst-performing island (blue). Livestock were also either tagged post-vaccination (black circles) or not (white circles). For the majority of vaccination rates, vaccine allocations and tagging strategies, Grande Comore averted the lowest percentage of infections on average (grey boxes), and tagging livestock resulted in a greater number of infections averted across the archipelago. The violins show the percentage of infections averted across on each island for different annual vaccination rates, allocation methods and tagging strategies. The points and boxplots show the median and inter-quartile range for each scenario respectively. All metrics shown were based on 25,000 model simulations.
