# Supplementary material for: Effectiveness and equity of vaccination strategies against Rift Valley fever in a heterogeneous landscape
Source: PLoS Negl Trop Dis. 2025 Jul 28;19(7):e0013346. doi: 10.1371/journal.pntd.0013346 (PMC12316399; doi:10.1371/journal.pntd.0013346)
Supplement: S10 Fig — The fitted metapopulation model describing Rift Valley Fever virus infection in livestock on each island of the Comoros archipelago—Grande Comore (red), Mohéli (blue), Anjouan (green) and Mayotte (purple)—was simulated forward in time for 35 years from the end of the fitting period, June 2015, under a range of vaccination strategies. This was then compared with the model predicted number of infections without any vaccination over the same time period. Shown is the median (solid line) and 95% prediction interval (light area) of model predicted number of infections averted on each island for 10% and 15% of livestock vaccinated annually across the archipelago, optimal vaccine allocation across islands, and two livestock tagging strategies. The median and 95% prediction intervals were calculated from 1000 model simulations. (PDF) [file pntd.0013346.s014.pdf]

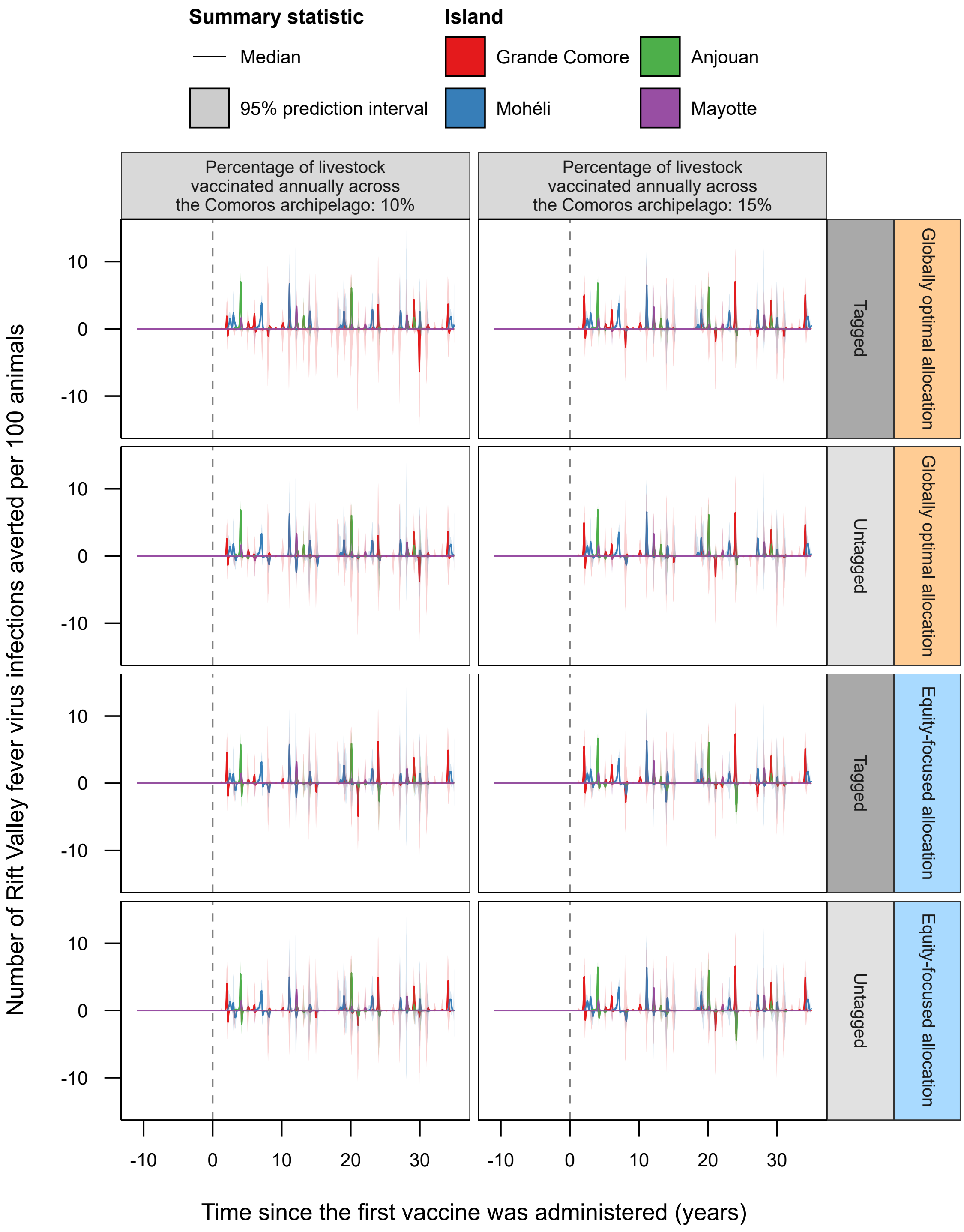

S10 Fig. Model predicted number of infected averted livestock per island in the Comoros archipelago with 10% and 15% vaccination rates. The fitted metapopulation model describing Rift Valley Fever virus infection in livestock on each island of the Comoros archipelago—Grande Comore (red), Mohéli (blue), Anjouan (green) and Mayotte (purple)—was simulated forward in time for 35 years from the end of the fitting period, June 2015, under a range of vaccination strategies. This was then compared with the model predicted number of infections without any vaccination over the same time period. Shown is the median (solid line) and 95% prediction interval (light area) of model predicted number of infections averted on each island for 10% and 15% of livestock vaccinated annually across the archipelago, optimal vaccine allocation across islands, and two livestock tagging strategies. The median and 95% prediction intervals were calculated from 1000 model simulations.
